# USP8‐Governed MDA5 Homeostasis Promotes Innate Immunity and Autoimmunity

**DOI:** 10.1002/advs.202503865

**Published:** 2025-06-17

**Authors:** Qimin Zhang, Shan Huang, Yan He, Weiwei Wang, Chao Tong, Mengru Ma, Manyu Zhao, Lian Yi, Klaus‐Peter Knobeloch, Peijing Zhang

**Affiliations:** ^1^ Department of Pharmacy Personalized Drug Therapy Key Laboratory Sichuan Academy of Medical Sciences & Sichuan Provincial People's Hospital University of Electronic Science and Technology of China Chengdu 610072 China; ^2^ National Engineering Research Center for Nanomedicine the Key Laboratory of Molecular Biophysics of Ministry of Education College of Life Science and Technology Huazhong University of Science and Technology Wuhan 430074 China; ^3^ Jiangxi Provincial People's Hospital The First Affiliated Hospital of Nanchang Medical College Nanchang 330006 China; ^4^ Department of Rheumatology and Immunology Union Hospital Tongji Medical College Huazhong University of Science and Technology Wuhan 430022 China; ^5^ Institute of Neuropathology University of Freiburg 79106 Freiburg Germany

**Keywords:** AKT, autoimmunity, deubiquitination, innate immunity, MDA5, USP8

## Abstract

The essential cytoplasmic RNA sensor Melanoma Differentiation‐Associated protein 5 (MDA5) initiates type I interferons (IFNs) signaling and subsequent immune responses. However, aberrant activation of MDA5 by viral infections or gain‐of‐function mutations leads to severe autoimmune diseases, for most of which effective treatment is limited. Here, it is shown that inactivation of ubiquitin‐specific protease 8 (USP8/UBPy) degrades the MDA5 protein, suppressing antiviral signaling and autoimmunity. It is found that viral infection modulates the AKT‐dependent phosphorylation of USP8 at serine 718, which not only promotes the activation of USP8 but also enhances the association between USP8 and MDA5 and the consequent deubiquitination and stabilization of MDA5. Inactivation of USP8 specifically degrades the MDA5 protein regardless of the mutation pattern. Genetic deletion of *Usp8* in mice contributes to decreased levels of type I interferons and proinflammatory cytokines. Importantly, inhibition of USP8 or AKT can effectively suppress MDA5‐induced autoimmunity in Aicardi–Goutières syndrome (AGS) mice and anti‐MDA5‐positive dermatomyositis (DM)/systemic lupus erythematosus (SLE) patient cells. Therefore, these results highlight the critical roles of USP8 in innate antiviral immunity against RNA viruses and autoimmunity and provide a potential therapy for treating autoimmune diseases associated with MDA5.

## Graphical abstract

This study reveals that USP8 stabilizes MDA5 via AKT‐mediated phosphorylation (Ser718), enhancing their interaction and MDA5 deubiquitination. USP8 inactivation degrades MDA5, suppressing type I IFN and cytokine production. Pharmacological inhibition of USP8/AKT alleviates MDA5‐driven autoimmunity, demonstrating the USP8‐MDA5 axis as a therapeutic target for autoimmune disorders linked to aberrant MDA5 activation.

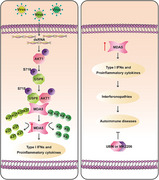

## 1 Introduction

Retinoic acid‐inducible gene RIG‐I‐like receptors (RLRs) serve as the host's primary response to danger. RLRs are a type of RNA helicase located in the cytoplasm of cells and include three members: RIG‐I, MDA5, and LGP2. They can recognize cellular viral RNA and activate the antiviral immune response. Upon binding RNA in the cytoplasm, MDA5‐RNA filaments recruit the downstream adaptor protein MAVS, which subsequently forms a CARD‐dependent helical filamentous structure. Filamentous MAVS recruits proteins from the TRAF and TRIM families to induce the expression of type I interferons and proinflammatory cytokines.^[^ ^1^, ^2^, ^3^ ^]^ MDA5 recognizes many viral RNAs, including some positive single‐stranded RNA viruses, such as encephalomyocarditis virus (EMCV), murine norovirus, poliovirus, and the picornavirus family. Recent reports have shown that SARS‐CoV‐2 plays dual roles in triggering the MDA5‐dependent interferon response and antagonizing the ISG15‐dependent activation of MDA5 through the papain‐like protease of SARS‐CoV‐2.

Dysregulation of MDA5 can lead to excessive enhancement of IFN signals, which, in turn, can cause autoimmune diseases. Approximately 30 distinct gain‐of‐function mutations in *IFIH1*, the gene that encodes MDA5, have been linked to autoimmune diseases in humans, such as AGS, Singleton‐Merten syndrome (SMS), SLE, DM, and type 1 diabetes. MDA5 mutations, depending on their position in the pathogenic map of MDA5, are known to be divided into three groups: mutations in the RNA binding domain, mutations in the ATP binding site, and mutations in binding sites other than the two. Mutations in the RNA binding domain disrupt immune tolerance by relaxing the RNA specificity of MDA5 and stabilizing the MDA5‐dsRNA filaments.^[^ ^4^, ^5^, ^6^ ^]^ In the other two cases, the consequence is to constrain conformation changes in MDA5 necessary for ATP hydrolysis and ATPase‐dependent dissociation from endogenous RNAs, along with increased IFNβ transcription.^[^ ^7^, ^8^, ^9^ ^]^ For example, the MDA5 mutant with substitution of serine for glycine at position 821 (G821S) (*Ifih1* ^gs/+^ or gs/+) can spontaneously develop lupus‐like nephritis and systemic autoimmune symptoms, indicating the complexity and difficulty of treating MDA5‐mediated autoimmune diseases. In addition to the genetic component,^[^ ^10^, ^11^ ^]^ environmental factors, such as viral infections, induce excessive activation of MDA5, which in turn causes autoimmunity.^[^ ^12^ ^]^Recent reports have shown that 48.2% of COVID‐19 patients are positive for anti‐MDA5 antibodies and that high titers of this antibody are associated with severe disease and an unfavorable prognosis.^[^ ^13^ ^]^ Thus, developing precision therapies that target the MDA5 protein to treat various MDA5‐mediated autoimmune diseases is urgently needed.

Post‐translational modifications, especially ubiquitination/deubiquitination, play a key role in immune signal transduction mediated by RLRs. A previous study demonstrated that knockdown of the non‐specific deubiquitinase USP17/DUB3 results in increased ubiquitination levels of MDA5 and RIG‐I, while total ubiquitination is also enriched. Thus, the specific deubiquitinating enzyme for MDA5 in viral infection and autoimmunity remains unknown.

USP8/UBPy is a member of the UBP family of deubiquitinating enzymes and plays an important role in regulating various physiological processes, including the immune response. Previous studies have shown that USP8 modulates viral infection by deubiquitinating caspase‐1 or IFNAR2.^[^ ^14^, ^15^ ^]^ Additionally, the specific knockout of Usp8 in mouse T cells can cause colitis, accompanied by a T‐cell imbalance in the intestines.^[^ ^16^ ^]^ Inhibition of USP8 also regulates T‐cell differentiation,^[^ ^17^ ^]^ prevents CD8+ T cells exhaustion,^[^ ^18^ ^]^ and enhances CD8+ T cells infiltration.^[^ ^19^, ^20^ ^]^ Additionally, Stress granule‐localized USP8 enhances cGAS‐mediated type I interferonopathy via deubiquitination of DDX3X.^[^ ^21^ ^]^ However, whether USP8 is involved in regulating the immune response induced by RLRs and autoimmunity has not been reported.

In this study, we provide evidence that USP8 interacts with, deubiquitinates, and stabilizes MDA5. Viral infection promotes the phosphorylation and activation of USP8 in an AKT‐dependent manner, which in turn increases the expression of MDA5 and, consequently, its antiviral ability. Blocking the activities of USP8 or AKT effectively suppressed MDA5‐induced autoimmunity in AGS mice and MDA5+ DM/SLE patient cells. These findings not only indicate that the AKT‒USP8 axis plays essential roles in innate immunity and autoimmunity but also provide a new perspective for the treatment of MDA5‐related autoimmune diseases.

## 2 Results

### 2.1 USP8 Dictates MDA5 Protein Homeostasis

**Figure 1:**
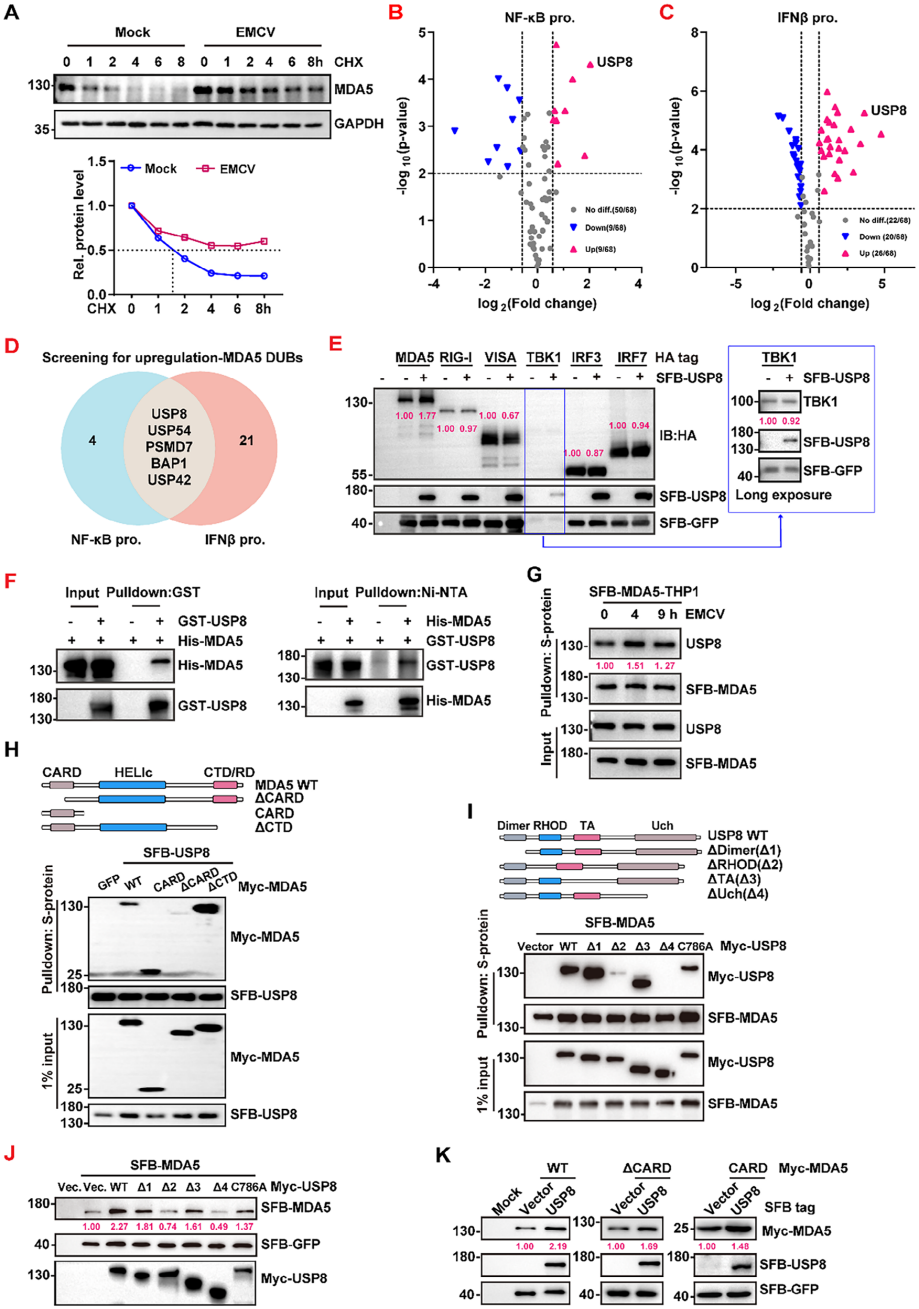
USP8 interacts with and upregulates the MDA5 protein. A) Immunoblot analysis of MDA5 and GAPDH (up panel) and quantification of the intensities of MDA5 (relative to GAPDH) (bottom panel) in THP1 cells infected with EMCV for 5 h followed by treatment with CHX for 0–8 h. B) Luciferase assay in HEK293T cells co‐transfected with the NF‐κB luciferase reporter (NF‐κB pro.), pRL‐TK, MDA5 CARD, or empty vector or DUBs for 24 h. Luciferase activities of DUBs on the NF‐κB luciferase reporter (Renilla luciferase as an internal control) were normalized to empty vector control activity. C) Luciferase assay in HEK293T cells co‐transfected with the IFNβ luciferase reporter (IFNβ pro.), pRL‐TK, MDA5, or empty vector or DUBs for 24 h. Luciferase activities of DUBs on the IFNβ luciferase reporter (Renilla luciferase as an internal control) were normalized to empty vector control activity. D) Venn diagram showing the overlapping DUBs that enhanced promoter's activity in the two screening assays described in (B) and (C). E) Immunoblot analysis of the MDA5, RIG‐I, VISA, TBK1, IRF3, IRF7 and SFB‐GFP in HEK293T cells transfected with empty vector or SFB‐USP8. F) Pull‐down analysis of the direct interaction between exogenous USP8 and MDA5 purified from BL21 *E. coli*. G) Pull‐down analysis of the interaction between endogenous USP8 and MDA5 in THP1 cells stably overexpressing MDA5 and infected with EMCV as indicated. H) Pull‐down analysis (with anti‐S protein) of the interaction between USP8 and wild‐type MDA5 or deletion mutants of MDA5 in HEK293T cells. I) Pull‐down analysis (with anti‐S protein) of the interaction between MDA5 and wild‐type USP8 (Myc‐USP8) or deletion mutants of USP8 in HEK293T cells. J) Immunoblot analysis of exogenous MDA5 in HEK293T cells transfected with full‐length SFB‐USP8 or truncated mutants. K) Immunoblot analysis of full‐length MDA5 or truncated mutants in HEK293T cells transfected with empty vector or SFB‐USP8. The data from the indicated wells per group are presented as the means ± SEMs. Each blot data is representative of three independent experiments. (B and C) *n* = 3 biologically independent experiments. P values were determined by t tests (B and C).

Upon EMCV infection, the half‐life of the MDA5 protein was significantly prolonged, suggesting that post‐translational modification but not transcriptional regulation plays an important role in MDA5 protein homeostasis (**Figure** **1**A; Figure S1A–C, Supporting Information). Thus, we assumed that targeting MDA5 deubiquitinases (DUBs) could lead to the destabilization of MDA5. To identify the MDA5 DUB, we conducted an unbiased screening of DUBs responsible for activating MDA5 signaling by using a panel of 68 DUBs fused to a triple‐epitope tag, SFB (S‐protein, FLAG tag, and streptavidin‐binding peptide), as previously established.^[^ ^22^ ^]^ The results demonstrated that overexpression of some candidate DUBs enhanced both NF‐κB and IFNβ promoter activities (Figure 1B,C). We considered DUBs to be significant if they had a *p* < 0.01 and log2 ^fold change^ > 0.58 (Tables S1 and S2, Supporting Information). Based on these criteria, we identified 9 DUBs that were identified as significant according to the NF‐κB promoter activity analysis (Figure 1D, blue pie graph) and 26 DUBs that were significant according to the IFNβ promoter activity analysis (Figure 1D, red pie graph). Among these DUBs, 5 DUBs (USP8, USP54, PSMD7, BAP1, and USP42) were significant in two screening assays (Figure 1D, dark brown oval graph). Given that USP8 ranked among the top two candidates in both screening assays, we prioritized USP8 for subsequent investigation. Consistently, USP8 overexpression significantly enhanced MDA5‐induced ISRE promoter activity (Figure S1D, Supporting Information). Collectively, our findings demonstrate that USP8 critically regulates MDA5 signaling.

We further found that USP8 specifically increased the protein level of exogenous MDA5, whereas RIG‐I, VISA (also known as MAVS, Cardif, and IPS‐1), TBK1, and IRF3/7 remained unaffected (Figure 1E), indicating that USP8 is a positive regulator of the MDA5 protein. We then examined the association between USP8 and MDA5 and found that USP8 specifically interacted with MDA5 but not with other related proteins, such as RIG‐I (Figure 1F; Figure S1E, Supporting Information), and that their association was potentiated after EMCV infection, but not Poly(I:C) HMW (Figure 1G; Figure S1F, Supporting Information). Domain mapping analysis demonstrated that the CARD domain of MDA5 formed the primary binding interface for USP8 (Figure 1H), while the RHOD and Uch domains of USP8 were responsible for its association with MDA5 (Figure 1I). Moreover, USP8 significantly increased the protein level of MDA5, which was abolished upon deletion of the RHOD and Uch domains or through mutation of the enzymatic activity site (Figure 1J). Additionally, the impact of USP8 on MDA5 persisted irrespective of whether the CARD or CTD domain was deleted (Figure 1K; Figure S1G, Supporting Information). Collectively, these findings suggest that USP8 plays a crucial role in regulating MDA5 protein homeostasis.

### 2.2 USP8 Deficiency Impairs EMCV‐Triggered Signaling In Vitro

**Figure 2:**
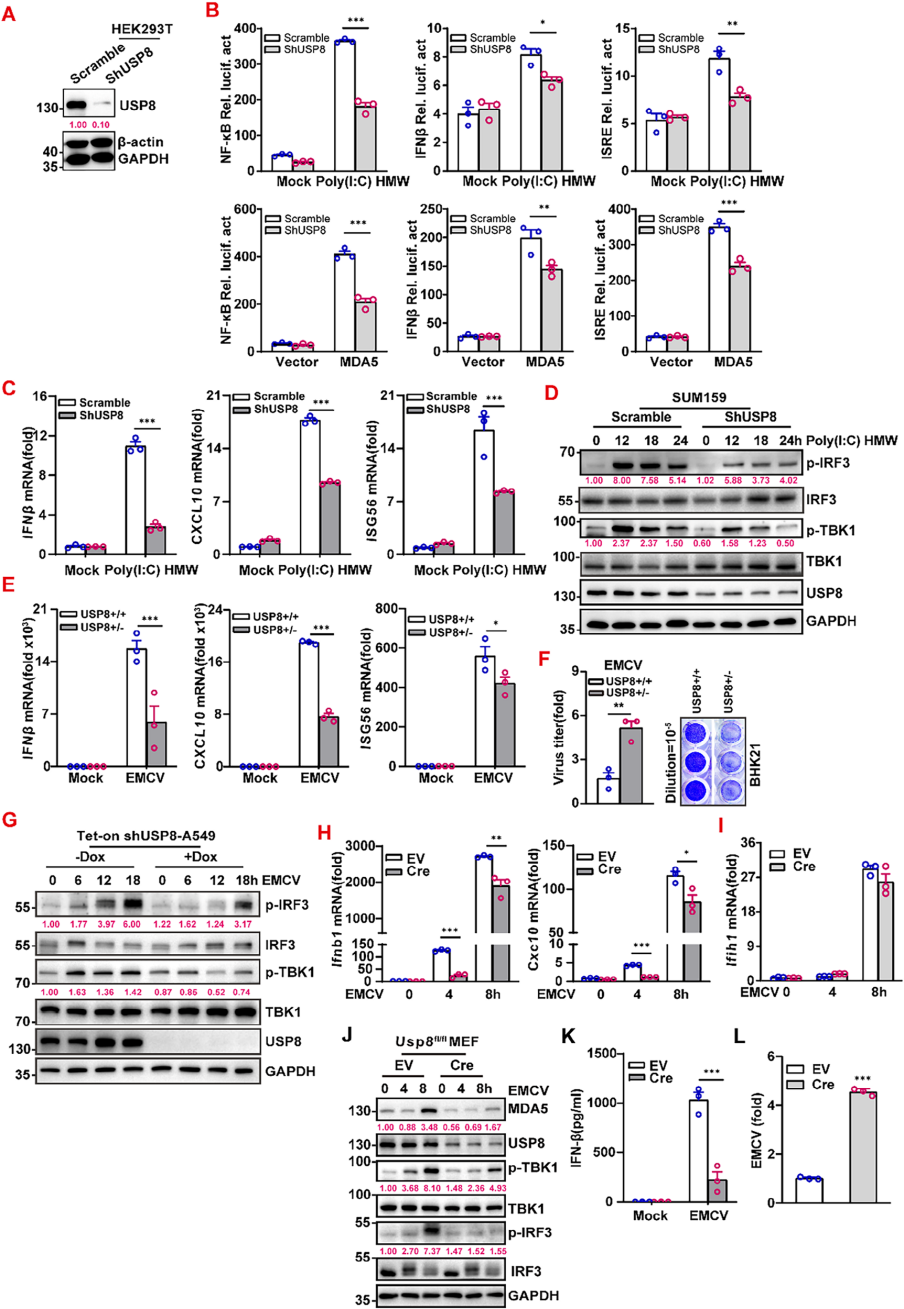
USP8 deficiency impairs the type I IFN antiviral immune response in vitro. A) Immunoblot analysis of the USP8 protein in USP8‐knockdown HEK293T cells. B) Luciferase assay of the NF‐κB, IFNβ, and ISRE luciferase reporter in a scramble or shUSP8‐HEK293T cells transfected with Poly(I:C) HMW or MAD5. **C)** RT‒qPCR analysis of *IFNβ*, *CXCL10*, and *ISG56* mRNA in a scramble or shUSP8‐SUM159 cells with or without Poly(I:C) HMW transfection. GAPDH was used as the internal reference. **D)** Immunoblot analysis of p‐IRF3, p‐TBK1, USP8, and GAPDH protein levels in scramble or shUSP8‐HEK293T SUM159 cells with or without Poly(I:C) HMW transfection at the indicated times. E) RT‒qPCR analysis of *IFNβ*, *CXCL10* and *ISG56* mRNA in USP8^+/+^ (sgGFP) or USP8^+/‐^ (sgUSP8) A549 cells with or without infection with EMCV. GAPDH was used as the internal reference. F) Viral titers of USP8^+/+^ or USP8^+/‐^ A549 cells infected with EMCV for 24 h were determined via a standard plaque assay. The cells were stained with crystal violet. G) Immunoblot analysis of p‐IRF3, p‐TBK1, USP8 and GAPDH protein levels in Tet‐on pLKO‐shUSP8‐ A549 cells treated ‐/+ Dox for 72 h followed by infecting with EMCV at the indicated times. H,I) RT‒qPCR analysis of *Ifnb1*, *Cxcl10* (H) and *Ifih1* (I) mRNA in *Usp8* ^fl/fl^ MEFs that were transduced for 48 h with control or Cre lentivirus and then infected with EMCV for 0–8 h. J) Immunoblot analysis of the MDA5, USP8, p‐TBK1, and p‐IRF3 proteins in *Usp8* ^fl/fl^ MEFs that were transduced and infected as described in (H). K,L) ELISA of IFN‐β K) from the supernatants of *Usp*8^fl/fl^ MEFs and RT‒qPCR analysis of EMCV replication L) in *Usp8* ^fl/fl^ MEFs that were transduced for 48 h with control or Cre lentivirus followed by infection with EMCV for 8 h. Data represent the analysis of the indicated *n* wells per group, means ± SEMs. Each blot data is representative of three independent experiments. (B, C, E, G, H, I and L) *n* = 3 biologically independent experiments; J) *n* = 3 independent experiments. P values were determined via two‐way ANOVA (B, C, E, I, and K), Multiple unpaired t‐tests H), Welch's *t*‐test F), and unpaired t‐tests L). ^*^ *p *< 0.05, ^**^ *p *< 0.01, ^***^ *p *< 0.001.

Because the association between USP8 and MDA5 was upregulated by EMCV infection, we explored the function of USP8 in the IFN response. Interestingly, USP8 knockdown significantly inhibited MDA5‐ and Poly(I:C) HMW‐induced NF‐κB, IFNβ, and ISRE promoter activity, respectively (**Figure** **2**A,B). Furthermore, USP8 knockdown attenuated the Poly(I:C) HMW‐induced expression of *IFNβ*, *CXCL10*, and *ISG56* in SUM159 cells (Figure 2C). In addition, USP8 knockdown substantially impaired the Poly(I:C) HMW‐induced phosphorylation of IRF3 and TBK1 (Figure 2D).

Considering that homozygous *Usp8* knockout mouse embryos are lethal, we used a heterozygous USP8 knockout cell line (USP8^+/‐^) generated via CRISPR/Cas9 for subsequent experiments. Consistently, USP8 deficiency significantly reduced EMCV‐induced expression of *IFNβ*, *CXCL10*, and *ISG56* in A549 cells (Figure 2E). As expected, the titer of EMCV in the supernatant of the USP8^+/‐^ cells was twofold greater than that in the control cells (Figure 2F). Furthermore, both EMCV‐induced phosphorylation of TBK1 and IRF3 were significantly reduced in Tet‐induced USP8 knockdown A549 cells (Figure 2G). Additionally, USP8 deficiency significantly reduced poly(I:C)‐induced expression of IFNβ and phosphorylation of TBK1 and IRF3 in A549 cells (Figure S2A,B, Supporting Information). These results indicate that the loss of USP8 limits the antiviral immune response in human cell lines.

To further validate these findings, we knocked down *Usp8* in *Usp8^fl/fl^* mouse embryonic fibroblast (MEF) cells via infection with Cre lentivirus and found that the EMCV‐induced expression of *Ifnb1* and *Cxcl10* and the EMCV‐induced phosphorylation of TBK1 and IRF3 were significantly impaired (Figure 2H–J). In addition, we found that the production of IFNβ was significantly decreased while the replication of EMCV was increased by fivefold in *Usp8*‐deficient MEFs (Figure 2K,L). Together, these results suggest that USP8 deficiency impairs EMCV‐triggered signaling in vitro.

### 2.3 USP8 Deficiency Increases Susceptibility to Lethal EMCV Infection

To investigate the physiological role of USP8 in response to EMCV infection in vivo, we generated Lyz2‐Cre *Usp8* ^fl/fl^ mice by crossing *Usp8* ^fl/fl^ mice with Lyz2‐Cre mice to delete USP8 specifically in macrophages. USP8 deletion was confirmed to be efficient via PCR and Western blotting (Figure S3A, B, Supporting Information). Flow cytometry analysis revealed no significant differences in the abundances of granulocytes, macrophages, CD4+ T cells, CD8+ T cells (except in the bone marrow), or B cells (except in the bone marrow) in the bone marrow and spleen between *Usp8* ^fl/fl^ and Lyz2‐Cre *Usp8* ^fl/fl^ mice (Figure S3C, D, Supporting Information). These findings suggest that USP8 myelogenous deficiency does not affect the development or homeostasis of major immune cell populations.

**Figure 3:**
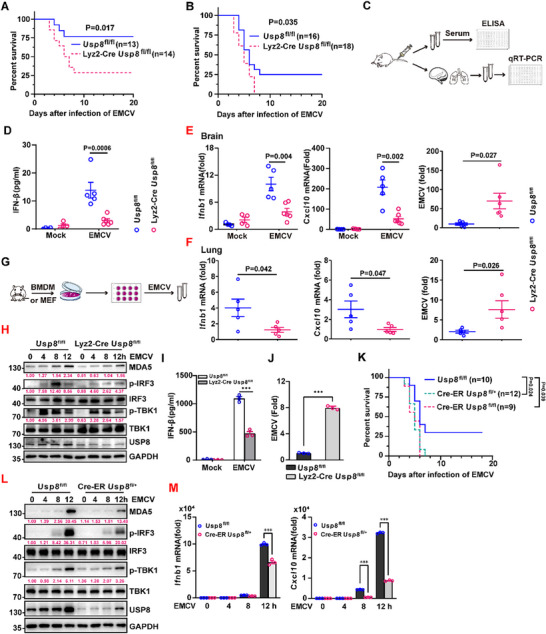
USP8 deficiency impairs the type I IFN antiviral response in vivo. A,B) Survival of age‐ and sex‐matched *Usp8* ^fl/fl^ and Lyz2‐Cre *Usp8* ^fl/fl^ mice after intranasal instillation of EMCV (1x10^14^ TCID_50_ per mouse) A) or intraperitoneal infection with EMCV (5x10^12^ TCID_50_ per mouse) B). C) Flow diagram of the experiment in (D, E). D) ELISA analysis of IFN‐β in the sera of *Usp8* ^fl/fl^ and Lyz2‐Cre *Usp8* ^fl/fl^ mice infected for 48 h via intraperitoneal injection of phosphate‐buffered saline (PBS) or EMCV (2x10^14^ TCID_50_). E,F) RT‒qPCR analysis of *Ifnb1* and *Cxcl10* mRNA and EMCV RNA in the brains E) and lungs F) of the mice described in (D). G) Flow diagram of the experiment in (G‐J). H) Immunoblot analysis of the MDA5, USP8, p‐TBK1 and p‐IRF3 proteins in BMDMs (*Usp8* ^fl/fl^ or Lyz2‐Cre *Usp8* ^fl/fl^) infected with EMCV for the indicated times. I) ELISA analysis of IFN‐β in the supernatants of BMDMs infected with EMCV for 12 h. J) RT‒qPCR analysis of EMCV replication in BMDMs infected with EMCV for 12 h. K) Survival of age‐ and sex‐matched Usp8^fl/fl^, Cre‐ER *Usp8* ^fl/+^ and Cre‐ER *Usp8* ^fl/fl^ mice intraperitoneally injected with tamoxifen (80 µg g^−1^ dissolved in corn oil) for five consecutive days and intravenously injected with EMCV (5x10^12^ TCID_50_ per mouse) 7 days later. L) Immunoblot analysis of the MDA5, USP8, p‐TBK1, and p‐IRF3 proteins in *Usp8* ^fl/fl^ and Cre‐ER *Usp8* ^fl/fl^ MEFs treated with 4‐OH tamoxifen followed by infection with EMCV for 0–12 h. M) RT‒qPCR analysis of *Ifnb1* and *Cxcl10* expression as described in (K). The data from the indicated wells per group are presented as the means ± SEMs. Each blot data is representative of three independent experiments. D,E) *n* = 4 in Mock group, *n* = 5 of *Usp8* ^fl/fl^ in EMCV group and *n* = 6 of Lyz2‐Cre *Usp8* ^fl/fl^ in EMCV group; F) *n* = 5 per group; I) *n* = 3 independent experiments; J,M) *n* = 3 biologically independent experiments. A,B, and K) *n* as shown in the figure. *p* values were determined via the log‐rank (Mantel‒Cox) test A,B, and K), two‐way ANOVA D,I, and M), Multiple unpaired t tests (E and F), upaired t‐tests J). ^*^ *p *< 0.05, ^**^ *p *< 0.01, ^***^ *p *< 0.001.

However, Lyz2‐Cre *Usp8* ^fl/fl^ mice died earlier and were more susceptible to EMCV infection than control *Usp8* ^fl/fl^ mice were (**Figure** **3**A,B). Consistently, the level of IFNβ in the sera of Lyz2‐Cre *Usp8* ^fl/fl^ mice was significantly lower than that in the sera of control littermates (Figure 3C,D). In addition, the expression of *Ifnb1* and *Cxcl10* was severely impaired, whereas EMCV replication was exacerbated in the brains or lungs of Lyz2‐Cre *Usp8* ^fl/fl^ mice compared with control littermates 48 h after EMCV infection (Figure 3E,F). Moreover, compared with control *Usp8^fl/fl^* BMDMs, USP8 deficiency decreased EMCV‐induced MDA5 protein levels, phosphorylation of TBK1 and IRF3, and IFNβ production but increased EMCV replication in Lyz2‐Cre *Usp8* ^fl/fl^ BMDMs (Figure 3G–J). These findings suggest that USP8 deficiency impairs EMCV‐triggered signaling in BMDMs.

To further validate the function of USP8, we generated Cre‐ER *Usp8* ^fl/+^ mice by crossing *Usp8* ^fl/fl^ and ROSA26‐CreERT2 mice (here referred to Cre‐ER). Usp8 can be deleted by intraperitoneal injection of tamoxifen (TAM). The survival rate of Usp8‐deficient mice exposed to EMCV was significantly lower than that of control *Usp8* ^fl/fl^ mice (Figure 3K). Usp8 deficiency also impaired EMCV‐induced increases in MDA5 protein levels and the phosphorylation of TBK1 and IRF3 in MEFs (Figure 3L). Similarly, the expression of *Ifnb1* and *Cxcl10* was significantly lower in Cre‐ER *Usp8* ^fl/+^ MEFs than in control *Usp8^fl/fl^* MEFs (Figure 3M). These results suggest that USP8 physiologically regulates EMCV‐induced production of type I IFNs and the expression of downstream genes and is necessary for the host's innate immune response to RNA viruses.

### 2.4 The DUB Activity of USP8 is Essential for Stabilizing MDA5

**Figure 4:**
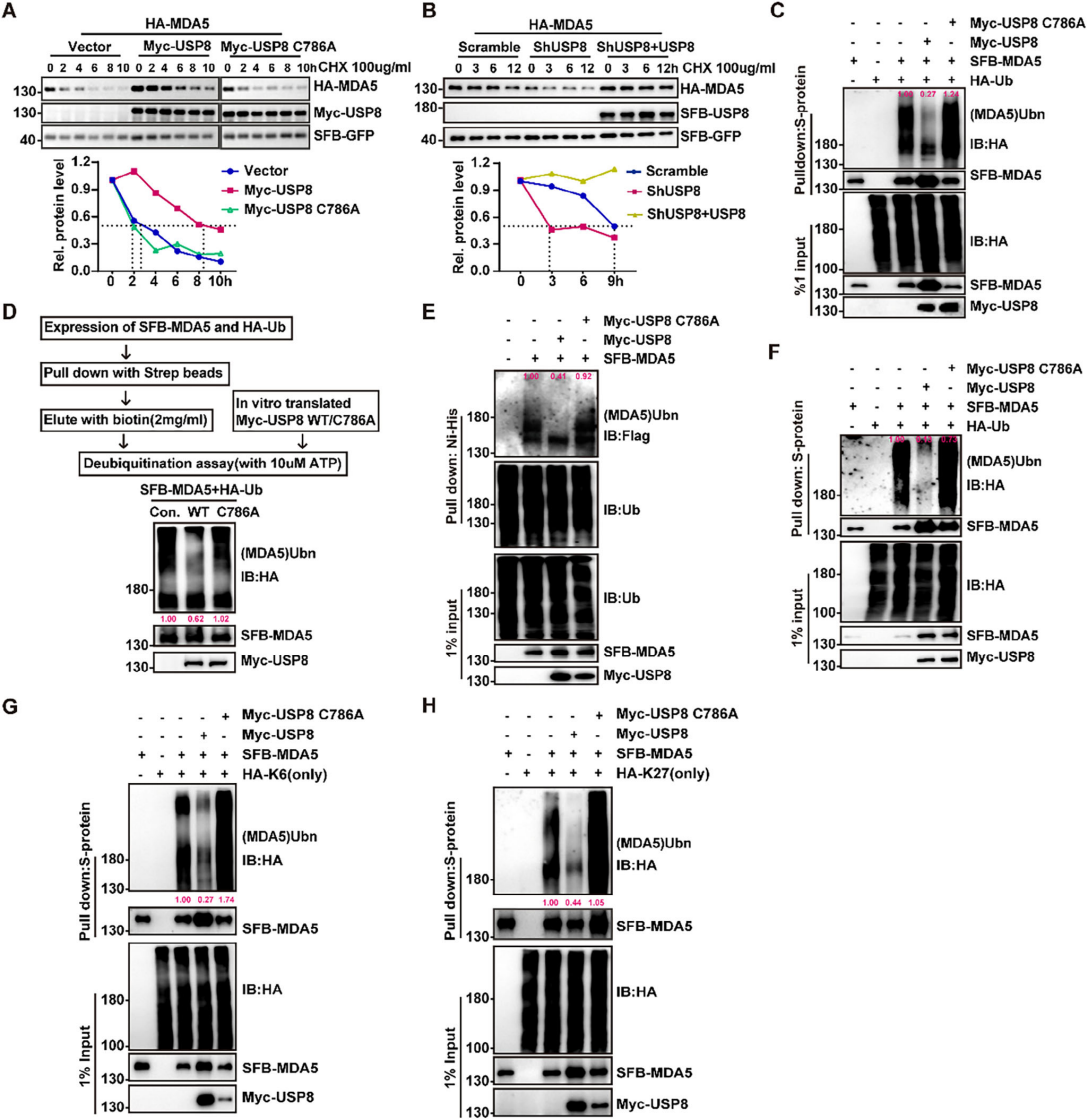
**USP8 deubiquitinates and stabilizes MDA5 through its catalytic activity. A)** Immunoblot analysis of SFB‐MDA5, Myc‐USP8, and SFB‐GFP (upper panel) and quantification of the intensities of SFB‐MDA5 (relative to SFB‐GFP) (lower panel) in HEK293T cells transfected with the control vector, WT Myc‐USP8 or the Myc‐USP8 C786A mutant in the presence of CHX for 0–9 h. B) Immunoblot analysis of HA‐MDA5, SFB‐USP8 and SFB‐GFP (left blots) and quantification of the intensities of HA‐MDA5 (relative to SFB‐GFP) (right graphs) in scramble HEK293T cells or USP8‐knockdown HEK293T cells reconstituted with vector or SFB‐USP8 in the presence of CHX for 0–12 h. C) Denature pull‐down (with anti‐S protein) and immunoblot analysis (with anti‐Flag, anti‐HA or anti‐Myc) of HEK293T cells transfected with SFB‐MDA5, HA‐Ub, and empty vector, Myc‐USP8 WT, or Myc‐USP8 C786A for 24 h. D) In vitro deubiquitination analysis of ubiquitin‐modified SFB‐MDA5 eluted from Streptavidin Sepharose by biotin (2 mg mL^−1^) incubated with in vitro‐translated USP8 WT or USP8 CA generated from an in vitro transcription and translation kit. E) Ni‐His pull‐down and immunoblot analysis of exogenous MDA5. SFB‐MDA5, Myc‐USP8, and Myc‐USP8 CA were expressed in 6xHis‐Ub‐HEK293T stable cells, and ubiquitin conjugates were recovered on Nickel magnetic beads under denaturing conditions. The cells were treated with MG‐132 for 6 h prior to harvesting. **F)** Denature pull‐down (with anti‐S protein) and immunoblot analysis (with anti‐Flag, anti‐HA, or anti‐Myc) of USP8^‐^deficient HEK293T cells transfected with SFB‐MDA5, HA‐Ub, and empty vector, Myc‐USP8 WT, or Myc‐USP8 C786A for 24 h. G,H) Denature pull‐down (with anti‐S protein) and immunoblot analysis (with anti‐Flag, anti‐HA or anti‐Myc) of HEK293T cells transfected with SFB‐MDA5, HA‐Ub (K6 or K27 only) and empty vector, Myc‐USP8 WT, or Myc‐USP8 CA for 24 h. Each blot data is representative of three independent experiments.

As shown, the loss of USP8 decreased the expression of endogenous MDA5, whereas the level of *IFIH1* mRNA did not change (Figure S4A, Supporting Information), suggesting that USP8 regulates MDA5 at the post‐translational level. To investigate whether USP8 maintains the stability of MDA5, we performed a cycloheximide (CHX, a protein synthesis inhibitor) assay. We found that the overexpression of USP8, but not the catalytic‐dead mutation of the cysteine at position 786 to alanine (USP8 C786A), significantly increased the half‐life of the MDA5 protein (**Figure** **4**A). Conversely, USP8 knockdown reduced the half‐life of the MDA5 protein, which was rescued by restoring USP8 expression (Figure 4B).

Since the protein level of MDA5 induced by EMCV was inhibited by treatment with a USP8 inhibitor (U8IN, which inactivates USP8 enzymatic activity) in L929 cells (Figure S4B, Supporting Information), we sought to investigate whether USP8 could remove the polyubiquitin chain from MDA5. As expected, wild‐type (WT) USP8 but not the USP8 C786A mutant catalyzed the deubiquitination of MDA5 in HEK293T cells or in vitro cell‐free system (Figure 4C–E). Moreover, when WT USP8 or the USP8 C786A mutant was overexpressed in USP8‐knockout HEK293T cells, we found that only WT USP8 eliminated the ubiquitination of MDA5 (Figure 4F). Furthermore, WT USP8, but not the catalytically dead mutant, increased the EMCV‐induced expression of *IFNβ* but decreased the replication of EMCV in USP8‐deficient HEK293T cells (Figure S4C,D, Supporting Information). These results indicate that USP8 deubiquitinates and stabilizes MDA5 through its catalytic enzyme active site.

MDA5 can undergo K27‐, K33‐ and K48‐linked ubiquitination for degradation.^[^ ^23^, ^24^, ^25^ ^]^ To explore the type of polyubiquitin chain in which USP8 removed from MDA5, we conducted deubiquitination experiments. The results revealed that USP8 can remove K6‐ and K27‐linked polyubiquitin chains from MDA5 with or without the CARD but not other types of polyubiquitin chains (Figure S4E,F, Supporting Information). Additionally, USP8, but not the USP8 C786A mutant, catalyzed the removal of K6‐ and K27‐linked polyubiquitin chains from MDA5 (Figure 4G,H). We next sought to identify the ubiquitination sites. The USP8‐mediated stabilization of MDA5 and its specific cleavage of K6‐linked ubiquitin chains on MDA5 without CARD (Figure 1K; Figure S4F, Supporting Information) collectively indicate that catalytic lysine residues reside outside the canonical CARD domain. Through bioinformatics prediction and domain mapping analyses, we identified lysine residues K235, K498, K688, and K865 as critical sites for USP8‐regulated MDA5 deubiquitination (Figure S4G, Supporting Information). Collectively, these results suggest that the activity of USP8 plays a critical role in MDA5‐dependent antiviral signaling.

### 2.5 AKT Phosphorylates USP8 at Serine 718 and Controls its Enzymatic Activity

**Figure 5:**
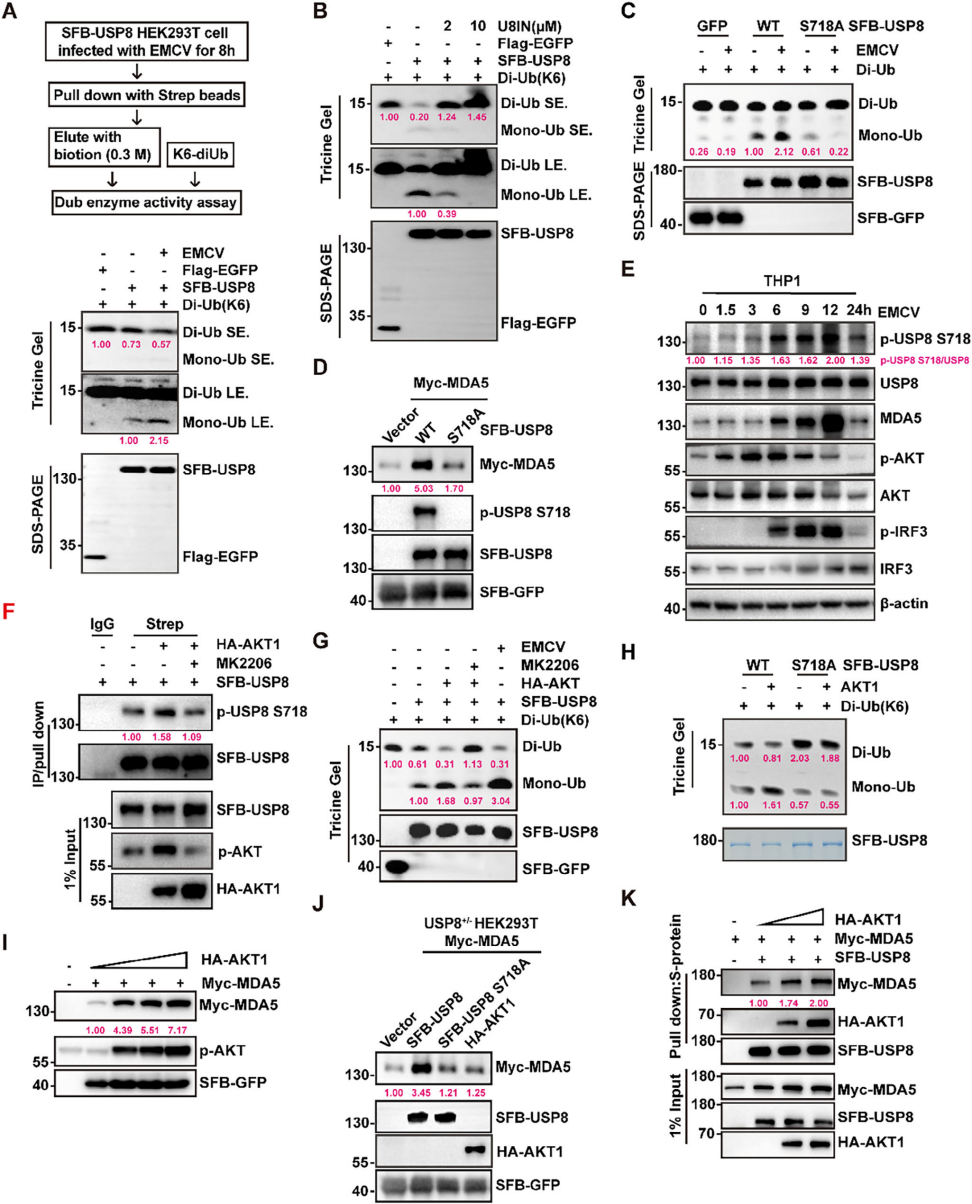
AKT1 phosphorylates and promotes the activity of USP8 and its association with MDA5. A) SFB‐USP8 was purified from cells infected with or without EMCV and incubated with K6‐linked Di‐ubiquitin at 37 °C for 40 min. B) SFB‐USP8 was purified and incubated with K6‐linked Di‐ubiquitin in the presence of DUB‐IN‐2 (USP8 inhibitor, U8IN) at the indicated dosages at 37 °C for 1 h. C) SFB‐USP8 and phosphorylation site mutants infected or not infected with EMCV were purified and incubated with K6‐linked Di‐ubiquitin in the presence of the compounds at 37 °C for 3 h. D) Immunoblot analysis of Myc‐MDA5 and SFB‐GFP in HEK293T cells transfected with empty vector, SFB‐USP8 WT, S718A, or S718D for 24 h. E) Immunoblot analysis of the phosphorylation levels of endogenous USP8 in THP1 cells infected with EMCV for 0–24 h. F) Immunoprecipitation (with Strep) and immunoblot analysis (with anti‐p‐USP8 Ser718) of the phosphorylation levels of USP8 in SFB‐USP8‐overexpressing HEK293T cells transfected with empty vector or HA‐AKT1 prior to treatment with MK2206. G) Purified SFB‐USP8 (co‐transfection with empty vector or HA‐AKT1 with or without MK2206) were incubated with K6‐linked Di‐ubiquitin in the presence of the compounds at 37 °C for 2 h. H) Purified SFB‐USP8 and S718A (co‐transfection with empty vector or HA‐AKT1) were incubated with K6‐linked Di‐ubiquitin in the presence of the compounds at 37 °C for 1 h. **()** Immunoblot analysis of exogenous protein levels of MDA5 in HEK293T cells overexpressing HA‐AKT1. J) Immunoblot analysis of Myc‐MDA5 and GAPDH protein expression in USP8^‐^deficient HEK293T cells transfected with empty vector, SFB‐USP8, SFB‐USP8 S718A or HA‐AKT1 for 24 h. K) Co‐immunoprecipitation analysis of the interaction between USP8 and MDA5 in HEK293T cells transfected with empty vector or the indicated dose of HA‐AKT1. Each blot data is representative of three independent experiments.

To further investigate how USP8 enzyme activity is regulated during viral infection, we performed an in vitro DUB assay. Interestingly, the ability of USP8 induced by EMCV infection to hydrolyze Di‐Ub was greater than that in the control group (**Figure** **5**A). Conversely, USP8 inhibitor treatment blocked the ability of USP8 to hydrolyze Di‐Ub (Figure 5B). These results suggest that the activity of USP8 can be regulated during EMCV infection.

Previous reports indicate that phosphorylation may regulate USP8 activity.^[^ ^26^, ^27^ ^]^ To determine whether EMCV infection activates USP8 through the phosphorylation of USP8, we performed co‐immunoprecipitation (Co‐IP) experiments. The results showed that EMCV infection dramatically induced the phosphorylation of USP8, as gauged by antibodies against pan‐phospho‐serine/threonine/tyrosine (Figure S5A, Supporting Information). To fully understand the underlying mechanism of EMCV‐induced activation of USP8, we determined the potential phosphorylation sites via mass spectrometry and identified three phosphorylation sites on USP8 (Serine 452/718 and Threonine 577) (Figure S5B, Supporting Information). We then generated USP8 mutants as indicated by the mutation of these amino acids to alanine (A). These mutants, along with WT USP8, were purified from HEK293T cells with or without EMCV infection, and their ability to hydrolyze Di‐Ub was tested. The results revealed that the USP8 S718A mutant lacked DUB activity (Figure 5C; Figure S5C, Supporting Information). Moreover, the EMCV‐induced activity of USP8 was completely abrogated when serine 718 was mutated to alanine (Figure 5C). Furthermore, the ubiquitination levels of MDA5 were decreased by WT USP8 but not the USP8 S718A mutant (Figure S5D, Supporting Information). Consistently, the protein levels of MDA5 were markedly increased by WT USP8 but not the phosphodeficient S718A mutant (Figure 5D). In addition, EMCV induced the phosphorylation of USP8 at S718 in a time‐dependent manner, which was tightly correlated with the induction of MDA5 protein expression (Figure 5E). These results indicate that EMCV infection activates MDA5 signaling by inducing the phosphorylation of USP8.

Previous studies have indicated that EMCV infection activates the PI3K‐AKT signaling pathway^[^ ^28^, ^29^, ^30^ ^]^ and that AKT‐mediated phosphorylation of murine USP8 at threonine 907 contributes to USP8 stability.^[^ ^31^ ^]^ However, whether EMCV infection or AKT regulates the catalytic activity of the USP8 protein remains elusive. Consistently, EMCV infection induced the phosphorylation of AKT, as gauged by antibodies against phospho‐AKT S473 (Figure 5E). Interestingly, we found that EMCV infection enhanced the interaction of MDA5, AKT1, and USP8 (Figure S5A,E, Supporting Information) and overexpression of AKT1 promoted the phosphorylation of human USP8, which was reversed by MK2206 (an AKT1 inhibitor) treatment (Figure 5F). To investigate whether AKT contributes to the regulation of USP8 activity, we performed an in vitro DUB assay using Di‐Ub. Like EMCV infection, AKT1 overexpression significantly increased WT USP8 enzymatic activity but not that of the USP8 S718A mutant, whereas MK2206 treatment completely reversed AKT1‐induced USP8 activity (Figure 5G,H). These results suggest that AKT activates USP8 by directly phosphorylating USP8 at serine 718.

As expected, the overexpression of AKT1 increased the protein level of exogenous MDA5 (Figure 5I). However, in USP8‐deficient HEK293T cells, overexpression of the AKT1 or USP8 S718A mutant did not increase the protein level of MDA5 compared with that of WT USP8 (Figure 5J). Consistently, MK2206 treatment inhibited the protein level of MDA5 and its downstream signaling in L929 cells infected with EMCV (Figure S5F, Supporting Information). Moreover, we found that AKT1 not only interacted with USP8 and MDA5 but also enhanced the interaction between USP8 and MDA5 in a dose‐dependent manner (Figure 5K; Figure S5G, Supporting Information). Taken together, these results suggest that AKT may phosphorylate USP8 to promote its activity and interaction with MDA5, thereby regulating MDA5 signaling.

### 2.6 Inactivation of USP8 or AKT Destabilizes MDA5 with Various Gain‐of‐Function Mutations

**Figure 6:**
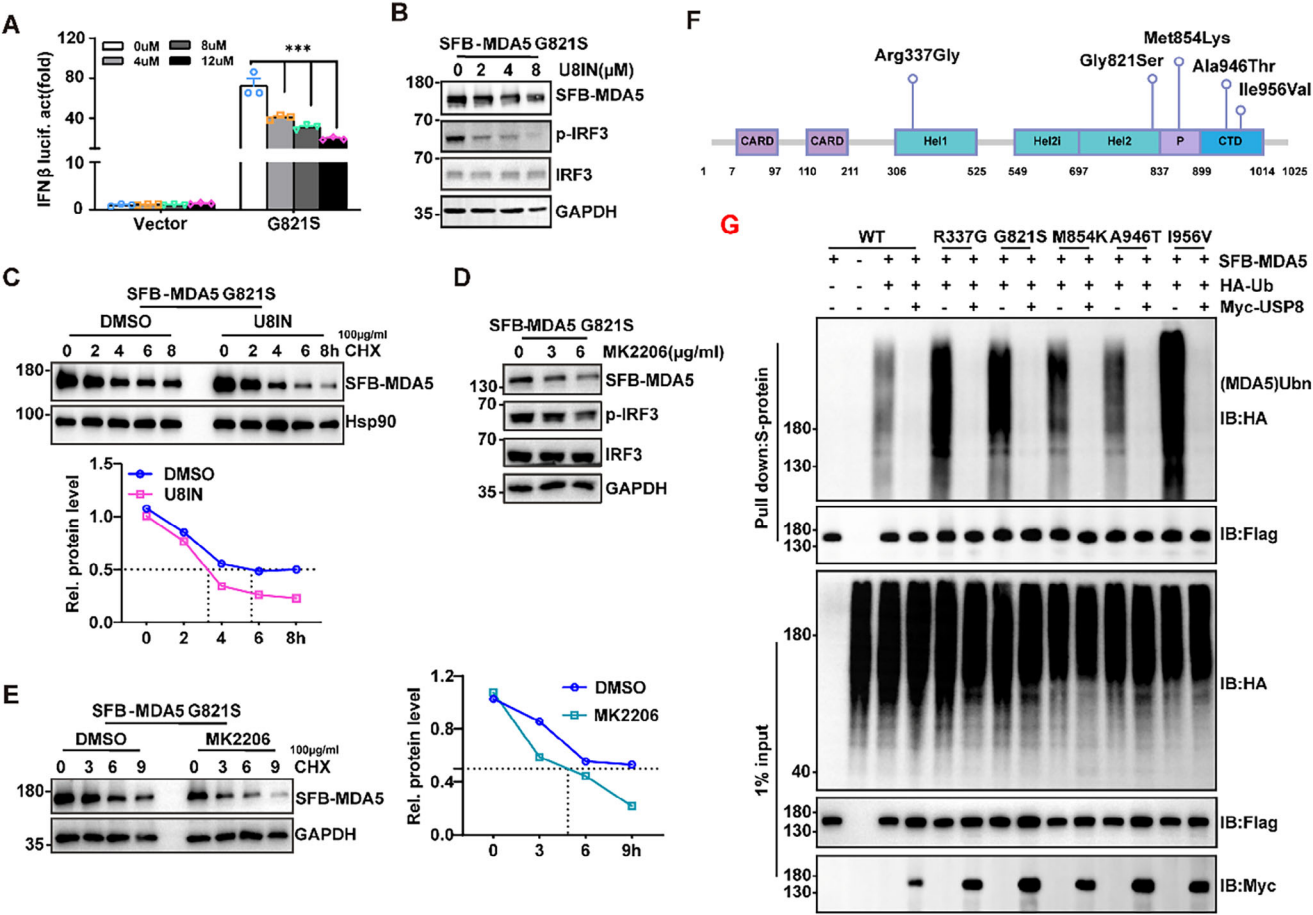
USP8 or AKT1 inhibition promotes the degradation of gain‐of‐function MDA5 mutants. A) Luciferase assay in HEK293T cells co‐transfected with the IFNβ luciferase reporter, pRL‐TK, and MDA5 G821S for 24 h, followed by treatment with U8IN for 9 h. Luciferase values were normalized to Renilla luciferase activity. B) Immunoblot analysis of the SFB‐MDA5 G821S, p‐IRF3, SFB‐MDA5 G821S, and GAPDH proteins in stable‐overexpressing‐SFB‐MDA5 G821S HEK293T cells treated with U8IN for 9 h. C) Immunoblot analysis of SFB‐MDA5 G821S and Hsp90 (top panel) and quantification of the intensities of SFB‐MDA5 G821S (relative to Hsp90) (bottom panel) in stable‐overexpressing‐SFB‐MDA5 G821S HEK293T cells treated with U8IN for 1 h followed by treatment with CHX for 0–8 h. **D**) Immunoblot analysis of the SFB‐MDA5 G821S, p‐IRF3, and GAPDH proteins in stable‐overexpressing SFB‐MDA5 G821S THP1 cells treated with MK2206 for 9 h. **E)** Immunoblot analysis of SFB‐MDA5 G821S and Hsp90 (left blots) and quantification of the intensities of SFB‐MDA5 G821S (relative to GAPDH) (right graphs) in stably overexpressing SFB‐MDA5 G821S HEK293T cells treated with MK2206 for 1 h followed by treatment with CHX for 0–8 h. **F)** Illustration of the *IFIH1* mutation sites. **G)** Denaturing immunoprecipitation (with anti‐S protein) and immunoblot analysis (with anti‐Flag, anti‐HA or anti‐Myc) of HEK293T cells transfected with SFB‐MDA5 (WT/R337G/G821S/M854K/A946T/I956V), HA‐Ub and empty vector or Myc‐USP8 for 24 h. Data represent the analysis of the indicated *n* wells per group, means ± SEMs. Each blot data is representative of three independent experiments. (A) *n* = 3 biologically independent experiments. *p* values were determined via two‐way ANOVA (A). ^***^ *p *< 0.001.

*IFIH1* mutations have been implicated in autoimmune diseases, such as the gain‐of‐function mutation MDA5 G821S, which triggers the aberrant production of type I IFNs, resulting in an autoimmune response.^[^ ^32^ ^]^ Because inactivation of AKT or USP8 destabilizes the wild‐type MDA5 protein and inhibits antiviral signaling, we hypothesize that targeting AKT or USP8 could counteract the excessive immune response induced by gain‐of‐function mutations in MDA5. As expected, overexpression of MDA5 G821S dramatically increased the activity of the IFNβ and ISRE promoters, as well as the phosphorylation of IRF3 (Figure S6A,B, Supporting Information). However, treatment with the USP8 inhibitor significantly attenuated the activity of the IFNβ promoter induced by MDA5 G821S in a dose‐dependent manner (**Figure** **6**A). Furthermore, the USP8 inhibitor markedly destabilized the MDA5 G821S protein, inhibited the phosphorylation of the IRF3 protein (Figure 6B), and disrupted the stability of the MDA5 G821S protein in stably overexpressing HEK293T cells (Figure 6C). Like treatment with the USP8 inhibitor, treatment with the AKT inhibitor also decreased the protein level of MDA5 G821S in THP1 cells and inhibited the MDA5 G821S‐induced phosphorylation of IRF3 (Figure 6D). Moreover, the AKT inhibitor strongly disrupted the stability of the MDA5 G821S protein in THP1 cells (Figure 6E). In addition, USP8 can remove polyubiquitin chains from MDA5 proteins associated with other pathogenic heterozygous mutations,^[^ ^33^ ^]^ such as R337G, M854K, A946T, and I956V (Figure 6F,G), and enhance their protein levels (Figure S6C, Supporting Information), indicating that the autoimmune response induced by various MDA5 mutations can be counteracted by the inhibition of USP8 activity. Thus, these findings suggest that inactivation of USP8 and AKT impairs the stability of MDA5 gain‐of‐function mutants such as MDA5 G821S, which reduces IFN activity and prevents an excessive immune response.

### 2.7 Inhibition of the USP8‒AKT Axis Alleviates Autoimmune Diseases

**Figure 7:**
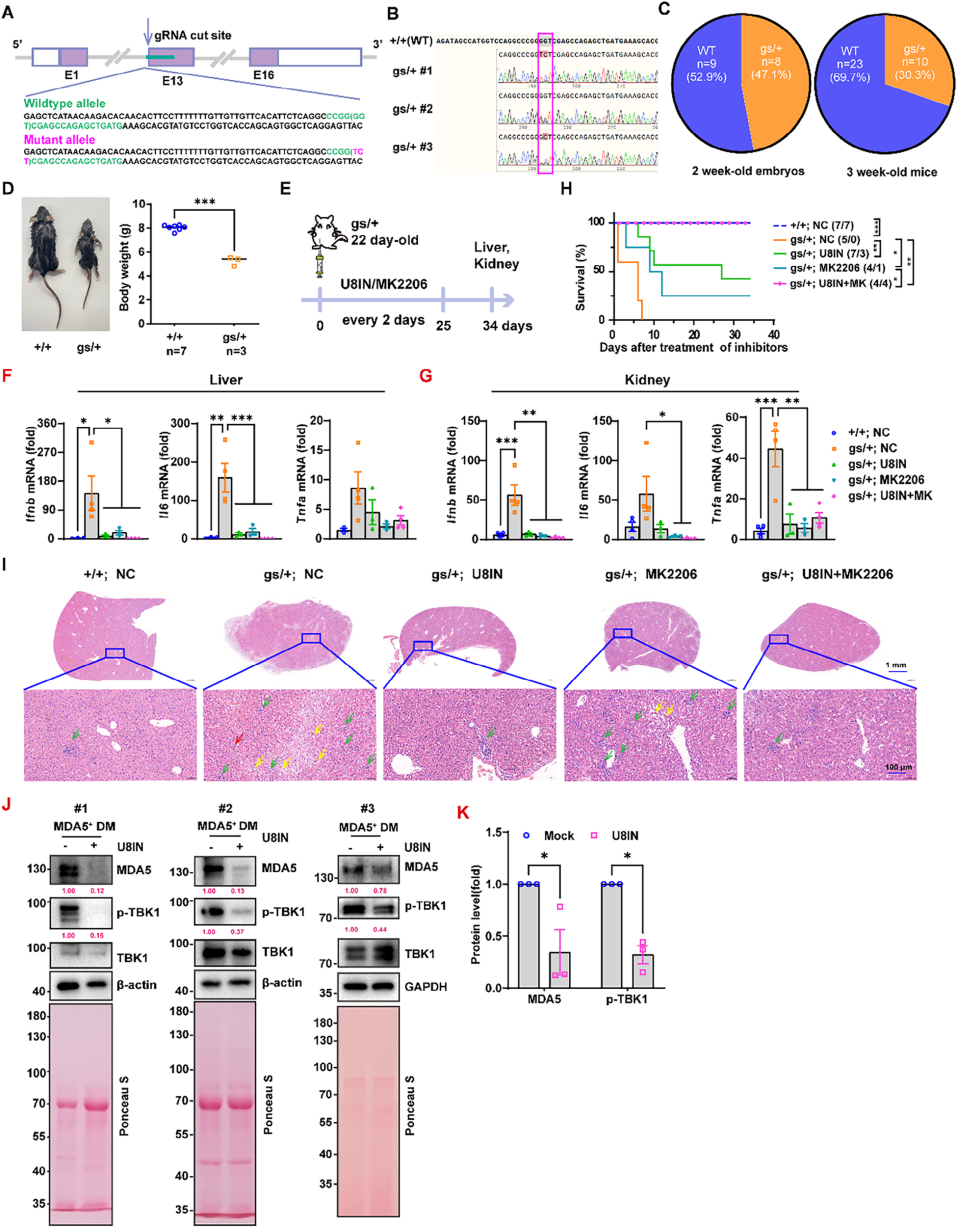
USP8 or AKT1 inhibition alleviates autoimmunity in AGS mice and anti‐MDA5‐positive DM/SLE patient cells. A) Illustration of CRISPR technology editing to generate G821S (gs/+) mutant mice. B) Sequencing identification of gs/+ mice. C) Genotyping analysis of embryos and mice at the indicated ages was performed from the interbreeding of WT and gs/+ mice. D) Body weights (right) and representative images (left) of WT and gs/+ mice. E) Flow diagram of the experiment in (F‐H). F–G) RT‒qPCR analysis of *Ifnb1*, *Tnfα* and *Il6* mRNA in the liver (F) or kidney (G) of the mice described in (E). H) Survival of age‐matched +/+ and gs/+ mice after intraperitoneal injection of U8IN or MK2206 alone or in combination. I) HE staining analysis of the livers treated as described in (E). Red arrow, increased cytoplasmic eosinophilic acid. Yellow arrow, hepatocyte steatosis, variously sized vacuoles in the cytoplasm. Green arrow, small focal aggregation of lymphocytes. J,K) Immunoblot analysis J) and normalized (K) of MDA5 and p‐TBK1 in PBMCs from patients treated with DMSO or the USP8 inhibitor for 16 h. Ponceau S staining for total protein normalization. Data represent the analysis of the indicated *n* wells per group, means ± SEMs. Each blot data is representative of three independent experiments. D) *n* = 7 in +/+ group, *n* = 3 in gs/+ group; (F) *n* = 3 in +/+, NC; gs/+, U8IN; gs/+, MK2206 group; *n* = 4 in gs/+, NC; gs/+, U8IN+MK2206 group; G) *n* = 4 in +/+, NC; gs/+, NC; gs/+, U8IN+MK2206 group; *n* = 3 in gs/+, U8IN; gs/+, MK2206 group; C,H) *n* as shown in the figure. *p* values were determined via unpaired t tests (D), one‐way ANOVA (F and G), log‐rank (Mantel‒Cox) tests H), and two‐way ANOVA K). ^*^ *p *< 0.05, ^**^ *p *< 0.01, ^***^ *p *< 0.001.

To determine whether USP8 or AKT inhibitor could be used to treat MDA5‐induced autoimmunity, we generated *Ifih1* ^gs/+^ (gs/+) mice via CRISPR editing (**Figure** **7**A,B) and found that the percentage of gs/+ mice was reduced from 47.1% (8 out of 17) of 2‐week‐old gs/+ embryos to 30.3% (10 out of 33) of 3‐week‐old gs/+ mice (Figure 7C). Similarly, the whole‐body weight of the gs/+ mice was significantly reduced by ≈35% (Figure 7D). As expected, USP8 or AKT inhibitor alone markedly inhibited the MDA5 G821S protein level in the gs/+ MEFs, whereas the combination of these two inhibitors had a synergistic inhibitory effect on the protein level of MDA5 G821S (Figure S7A, Supporting Information). The inhibition of USP8 or AKT activity subsequently suppressed the expression of *Ifnb1*, *Tnfα* and *Il6* in the gs/+ MEFs, and the combination of treatments was more effective (Figure S7B, Supporting Information). Further in vivo results revealed that the inhibition of USP8 or AKT could reduce the expression of *Ifnb1*, *Tnfα* and *Il6* (except in the liver) mRNAs in the liver and kidney of gs/+ mice (Figure 7E–G), leading to an increase in their survival rate (Figure 7H). Moreover, the combined administration of both inhibitors significantly increased the survival rate of the mice, reaching 90% (Figure 7H). In addition, the USP8 inhibitor effectively reversed the fatty liver phenomenon in gs/+ mice (Figure 7I). These findings highlight the potential of USP8 or AKT1 inhibition as a promising therapeutic strategy for MDA5‐associated AGS.

Anti‐MDA5‐positive dermatomyositis (MDA5‐DM) is also an autoimmune disease that tends to be complicated by rapidly progressive interstitial lung disease (RP‐ILD) with a very high mortality rate.^[^ ^34^, ^35^ ^]^ In addition, gain‐of‐function polymorphisms of *IFIH1* are associated with increased type I IFN signaling and an increased risk of SLE.^[^ ^36^ ^]^ To further assess the effect of a USP8 inhibitor on the treatment of MDA5‐related autoimmune disease, we recruited 4 anti‐MDA5‐positive patients (3 MDA5‐DM patients and 1 anti‐MDA5‐positive SLE patient) and isolated their Peripheral blood mononuclear cells (PBMCs). Interestingly, treatment with the USP8 inhibitor significantly reduced the protein levels of MDA5 and p‐TBK1 in PBMCs (Figure 7J,K; Figure S7C, Supporting Information), suggesting that USP8 could be a promising therapeutic target for treating MDA5‐related autoimmune diseases. Meanwhile, we found a positive correlation between the phosphorylation levels of USP8 and AKT in PBMCs of anti‐MDA5‐positive patients (*R^2^* = 0.8823, *p* = 0.0303) (Figure S7D,E, Supporting Information), indicating the AKT‐USP8 regulatory axis occurred in patients.

## 3 Discussion

The RNA sensor MDA5 critically mediates the innate antiviral immune response and autoinflammatory diseases.^[^ ^8^, ^37^ ^]^ The activity and availability of MDA5 are inversely regulated by ubiquitination and deubiquitination to elicit antiviral immunity while avoiding excessive autoimmunity.^[^ ^38^, ^39^ ^]^ However, there is currently no specific DUB for deconjugating polyubiquitin chains from MDA5, particularly in autoimmune diseases. In this study, we found that USP8 strongly interacts with and removes polyubiquitin chains from MDA5, which stabilizes MDA5 to activate the type I IFN response. USP8 deficiency significantly inhibits sustained IFN production induced by functionally enhanced MDA5 mutants, which in turn ameliorates MDA5‐associated type I interferonopathy (Figure S8, Supporting Information).

In support of this notion, we discovered that USP8 binds to MDA5 and VISA but specifically increases the protein level of exogenous MDA5. USP8‐deficient cells retained lower protein levels of MDA5 with or without EMCV infection, whereas the restoration of USP8 blocked the reduction in the MDA5 protein level. Furthermore, USP8‐deficient mice were more susceptible to EMCV infection than their wild‐type littermates were. K63‐linked polyubiquitination of the CARD domain of MDA5 promotes its autophagic degradation.^[^ ^39^ ^]^ Interestingly, we found that USP8 can inhibit the polyubiquitination of MDA5 regardless of the presence of the CARD. In brief, these findings reveal specific roles of USP8 in the innate antiviral immune response and autoimmune diseases via the deubiquitination and stabilization of MDA5.

Previous studies have indicated that PI3K‐AKT signaling may be activated after EMCV infection,^[^ ^28^, ^29^, ^30^ ^]^ but the mechanisms by which AKT regulates the activation of IRF3‐mediated expression of IFNβ remain elusive. Here, we found that AKT functions upstream of USP8 to regulate its phosphorylation, confirming a role for AKT in the modulation of USP8 catalytic activity in MDA5 signaling upon EMCV infection. Conflicting studies have revealed that the activity of USP8 may be inhibited or increased by its phosphorylation. Although USP8 can be phosphorylated,^[^ ^27^, ^31^, ^40^, ^41^, ^42^ ^]^ whether and how this phosphorylation affects the activity of USP8 is not very clear. Our study revealed that AKT can phosphorylate human USP8 at Ser718. More importantly, EMCV infection can promote the phosphorylation of Ser718 and consequently increase the activity of USP8 to stabilize the protein levels of MDA5, which ultimately activates the antiviral immune response.

In addition to antiviral surveillance, MDA5 has been implicated in autoimmune diseases such as SLE and AGS. As mentioned, the excessive activation of MDA5 caused by the G821S mutation results in autoimmune triggering associated with the aberrant production of type I IFNs. Since G821S MDA5 is negative for ATPase activity, few strategies exist to block the activity of mutated MDA5. Interestingly, the inhibition of USP8 or AKT significantly destabilizes MDA5 G821S, resulting in a decrease in IFN activity. Moreover, inactivation of USP8 significantly decreases the protein levels of MDA5 and p‐TBK1 in PBMCs isolated from DM/SLE patients, suggesting the potential of USP8 inhibitors for the treatment of these diseases. Recent studies have reported that mutations in the Z‐RNA recognition motif of the RNA‐editing enzyme ADAR1, which is an upstream receptor of MDA5, are associated with failure to edit a subset of self‐RNAs. These self‐RNAs can cause autoimmunity by activating MDA5.^[^ ^43^, ^44^, ^45^ ^]^ It is also worth testing whether inhibitors that target USP8 or AKT can reduce the protein level of MDA5 to treat autoimmunity related to the ADAR1‒MDA5 axis.

Taken together, our findings not only reveal the detailed mechanism by which the MDA5 protein homeostasis is modulated but also suggest that inactivation of USP8 or AKT may be an effective strategy for the treatment of autoimmunity related to MDA5, regardless of the type of mutation.

## 4 Experimental Section

### Cell Lines and Cell Culture

THP‐1 cells were cultured in RPMI 1640 medium supplemented with 10% FBS and 1% penicillin and streptomycin; L929 (NCTC clone 929; CL‐0137), BHK‐21 (CL‐0034, Procell) cells were cultured in MEM supplemented with 10% FBS and 1% penicillin and streptomycin according to the instructions; and HEK293T, SUM159 and A549 cells were cultured in DMEM supplemented with 10% FBS and 1% penicillin and streptomycin. Bone marrow cells were harvested from the tibias and femurs of 8‐week‐old C57BL/6 mice. For the generation of BMDCs, bone marrow cells were cultured in a complete medium containing GM‐CSF (10 ng mL^−1^) and IL4 (10 ng mL^−1^) for 7 days. For the generation of BMDMs, bone marrow cells were cultured in a complete medium containing 30% medium conditioned with L929 cells. Stable overexpression, knockdown, or knockout HEK293T, SUM159, and A549 cells were generated via infection with lentivirus‐based open reading frames (ORFs), shRNAs or sgRNAs (sequences used for each construct are listed in Tables S5 and S6, Supporting Information).

### Mice

*Usp8* ^fl/fl^ mice were generated as described in a previous study^[^ ^16^, ^46^ ^]^ and bred to Lyz2‐Cre C57BL/6 mice to generate myeloid‐specific USP8 knockout mice or to Cre‐ER (B6.129‐Gt‐(ROSA) 26Sor^tm1(cre/ERT2)Tyj^) mice to generate tamoxifen‐induced conditional USP8‐deficient mice (PCR primers for genotyping are listed in Table S5, Supporting Information). Lyz2‐Cre C57BL/6 mice were obtained from Dr. Tao Li (National Center of Biomedical Analysis). Cre‐ER (B6.129‐Gt‐(ROSA) 26Sor^tm1(cre/ERT2)Tyj^) mice were obtained from Dr. Bo Zhong (Wuhan University). The *Ifih1* ^gs/+^ mice with point mutations (G821S), obtained from Cyagen Biosciences, were generated via CRISPR/Cas‐mediated genome engineering. Both male and female mice were used for all the experiments. All the mice were bred under specific pathogen‐free (SPF) conditions, and all the experiments were performed in accordance with the guidelines approved by the Institutional Animal Care and Use Committee of Huazhong University of Science and Technology (approval number: S2927).

### Antibodies

The following primary antibodies were used in this study (or listed in Table S3, Supporting Information): Anti‐USP8 (27791‐1‐AP), Anti‐HA‐Tag (51064‐2‐AP), Anti‐Myc‐Tag (GNI4110‐MC) and Anti‐GAPDH (60004‐1‐Ig) from Proteintech; Anti‐β‐actin (SC‐47778); Anti‐AKT (pan) (4691), Anti‐P‐AKT S473 (4060), Anti‐MDA5 (5321), Anti‐TBK1/NAK (3504), Anti‐p‐TBK1/NAK (S172) (5483), Anti‐IRF3 (4302), Anti‐p‐IRF3 (S396) (4947), Rabbit mAb IgG XP isotype control (3900), Mouse anti‐rabbit IgG (Conformation Specific) (L27A9) mAb (3878) from Cell Signaling Technology; Anti‐p‐Ser/Thr/Tyr (ADI‐905‐522‐1) from Enzo Life Sciences;Anti‐ubiquitin (07‐375) from Merck Millipore. Anti‐phosphor‐Serine718 hUSP8 antibody was generated by immunizing rabbits with a synthetic peptide of human USP8 (Cys‐REPSKLKRSYS(p)SPDI) by AtaGenix Laboratories Co., Ltd. (Wuhan), Wuhan, PR China.

### Chemicals

Poly(I:C) HMW (tlrl‐pic) was obtained from InvivoGen, and the final concentration during transfection was 2–6 µg mL^−1^ according to the cell type; Poly(I:C) was obtained from Sigma Aldrich, and the final concentration during transfection was 0.5–3 µg mL^−1^ according to the cell type; GM‐CSF (AF‐315‐03) and IL4 (AF‐214‐14) from PeproTech; S‐protein Agarose (69704) from Millipore; Anti‐Myc Agarose (A7470) from Sigma‐Aldrich; BeaverBeads™ IDA‐Nickel (70501‐5) from Beaver (Beaver); Ni‐NTA Agarose (1018244) from QIAGEN; GST‐tag Purification Resin (P2253) and Protein A+G Agarose (P2055) from Beyotime; Actinomycin D (HY‐17559) and Doxycycline (HY‐NO565) from MCE; MK2206 2HCl (1032350‐13‐2), MG132 (S2619) and Cycloheximide (CHX, S6418) from Selleck; and the USP8 inhibitor (MB7295) from Meilunbio. Other chemicals and recombinant proteins are listed in Table S4 (Supporting Information).

### Plasmid Construction and Transfection

The full‐length sequence of USP8 was obtained from the DUB plasmid library of our laboratory and inserted into Myc‐Destination and pcDNA3.1^+^ vectors via seamless cloning, and the mutant and truncated versions were inserted into the Myc‐Destination vector. The PRK‐Flag‐MDA5 and PRK‐HA‐MDA5/ΔCTD plasmids were gifts from Dr. Hongbing Shu (Wuhan University). The full‐length sequence MDA5 was subsequently inserted into the SFB‐Destination and Myc/His‐pcDNA3.1A‐ vectors via seamless cloning, and the mutant and truncated versions were subsequently inserted into the Myc/His‐pcDNA3.1A vectors. The EZ‐Tet‐pLKO‐Puro was a gift from Cindy Miranti (Addgene plasmid # 85966; RRID: Addgene_85966). All the plasmids were validated by sequencing. The primer sequences are shown in Table S1 (Supporting Information). The ISRE promoter‐luciferase and pRL‐TK plasmids were gifts from Dr. Yibing Zhang (Institute of Hydrobiology, Chinese Academy of Science). The plasmids were transfected into HEK293T cells using PEI and into other cells via Lipo8000TM (C0533, Beyotime Biotechnology).

### Lentiviral Transduction

HEK293T cells seeded and cultured overnight in 10 cm dishes were used for lentiviral packaging. The pLKO‐USP8‐shRNA, EZ‐Tet‐pLKO‐USP8‐shRNA‐Puro, Lenti‐Cas9‐USP8‐sgRNA, Lenti‐SFB‐MDA5, and control vectors were transfected into HEK293T cells with the lentiviral packaging plasmids psPAX2 and pMD2.G, respectively. The transfection medium was replaced with fresh medium after 6 h. After 72 h of cell transfection, the supernatant of the cell culture was harvested and filtered with a 0.45 µm filter. The filtrate containing the lentivirus was directly used to infect cells or stored in a −80 °C freezer. HEK293T, SUM159, A549, and THP1cells seeded and cultured overnight in 6‐well plates were infected with lentiviral supernatants, and the medium was changed to fresh medium after 4 h. After 48–72 h of infection, the cells were selected with an appropriate concentration of puromycin for 4–7 day for western blotting or monoclonal screening in 96‐well plates.

### Viral Infection and Titer Tests

Cells seeded into 6‐well plates at 5–8 × 10^5^ cells per well or 96‐well plates at 4–5 × 10^4^ cells per well overnight were infected with a certain titer of EMCV. After 1 h, the medium was removed, the cells were washed twice with PBS, a fresh medium was added, and the cells were cultured for a period of time. The cells were harvested to detect target gene mRNA expression via RT‒qPCR (real‐time quantitative PCR) or to detect target protein expression via western blotting. The cell supernatant was harvested and diluted in a tenfold gradient to infect BHK‐21 cells. The CPE was recorded after the cells were cultured for 5‒7 days. USP8^fl/fl^ C57BL/6 mice and Lyz2‐Cre USP8^fl/fl^ C57BL/6 mice were injected with EMCV, and the living conditions were recorded every day. The lungs, livers, and spleens of the mice that were injected for 24 or 72 h were subjected to qRT‒PCR or virus titer tests. The EMCV virus was a gift from Dr. Hongbing Shu (Wuhan University).

### Plaque Assay

Virus‐infected cell supernatant or mouse tissue was diluted in a tenfold gradient to infect BHK‐21 cells. After 1 h, the medium was removed, the cells were washed twice with PBS, a fresh medium was added, and the cells were cultured for 48 h. The cells were then fixed with a 4% PFA solution for 15 min, stained with 1% crystal violet for 30 min, and washed with PBS 3 times. Plaques were recorded and photographed.

### Luciferase Reporter Assay

HEK293T cells seeded in 48‐well plates and cultured at 37 °C overnight were transfected with 0.025 µg pRL‐TK and 0.1 µg NF‐κB, IFNβ or ISRE luciferase promoter reporter plasmids together with 0.2 µg DUB expression plasmids. The cells were harvested after being cultured for 48 h or infected with a certain titer of EMCV after being cultured for 24 h. After 12 h of infection, the cells were harvested and used to measure luciferase activity according to the instructions. The luciferase activity was determined via a Dual‐Glo Luciferase Assay System (E2940).

### Real‐Time Quantitative PCR and ELISA

Total RNA was purified with a HiPure Total RNA Mini Kit (R4111‐03) and used to synthesize cDNA with a reverse transcription reagent kit (RR036A‐1 or A5001) according to the instructions. A QuantiNova SYBR Green PCR Kit (500) (208054) and a Bio‐Rad CFX Maestro system were used for qRT‒PCR. GAPDH was used as an internal control. Details of the qRT‒PCR primers used are given in Table S1 (Supporting Information). The levels of IFNβ in the serum and cell supernatant were measured with a Mouse IFN‐β Quantikine ELISA Kit (MIFNBO) according to the manufacturer's instructions.

### Flow Cytometry Analysis

Bone marrow was isolated from the tibiae and femurs of 8–10‐week‐old mice. Spleens from 8 to 10‐week‐old mice were mashed through a 70 µm cell strainer (Biologix Falcon) to yield single‐cell suspensions. Cells were counted and washed with PBS before being incubated with Zombie Aqua dye (77413) diluted 1:500 in PBS for 25 min at room temperature. The cells were washed once with 500 µL of FACS buffer (PBS containing 1% BSA and 2 mm EDTA) and then stained with TruStain FcX (antimouse CD16/32, 101320) in FACS buffer for 10 min in the dark at 4 °C. The cells were washed once with 500 µL of FACS buffer and then stained with surface antibodies against CD45 (103132), CD3 (100218), CD8α (100707), CD4 (100510), CD11b (101212), Ly6G/Ly6C (108408) and CD19 (115505) in FACS buffer for 30 min in the dark at 4 °C. The cells were washed once with 500 µL of FACS buffer and analyzed via Cytoflex LX (Beckman) and CytExpert software 2.3.

### Isolation of PBMCs From Patients

After written informed consent was obtained, we collected the peripheral blood of patients with the help of the Department of Rheumatology and Immunology, Union Hospital, Tongji Medical College of Huazhong University of Science and Technology. Human blood samples were obtained following the National Institutes of Health Guidelines for the use of human samples and with the approval of the Scientific Investigation Board of Tongji Hospital of Huazhong University of Science and Technology (approval number: S170). Human primary PBMCs were isolated with lymphocyte separation medium (human) (P8610) according to the manufacturer's protocols. The cells were maintained in RPMI‐1640 medium supplemented with 10% FBS, 2 mm glutamine, 1% penicillin and 1% streptomycin.

### Phosphorylation Mass Spectrometry Analysis

Stable expressing SFB‐USP8 HEK293T cells were harvested and lysed. The supernatant was collected, and Strep beads were incubated to capture the SFB‐USP8 protein complex. The eluent was obtained through biotin competitive elution, followed by incubation with S‐protein beads. The beads were then collected, and 2x sample buffer was added before heating at 95 °C for 8 min. The proteins were separated via SDS‒PAGE and stained with Coomassie bright blue. The target band was excised for in‐gel enzymatic digestion, followed by LC‒MS/MS analysis and data processing, which were performed by Jingjie Biotechnology Co., Ltd.

### Co‐Immunoprecipitation

The cells were harvested, and freshly prepared NP‐40 lysis buffer (20 mm Tris‐HCl, pH 8.0; 150 mm NaCl; 1% NP‐40; 10% glycerol; 2 mm EDTA, pH 8.0) supplemented with protease inhibitors was added. The mixture was incubated at 22 °C for 5 min to lyse the cells, and the mixture was centrifuged at 12000 rpm for 10 min at 4 °C. The supernatant was harvested and incubated with Anti‐Myc or S‐protein Agarose beads at 4 °C overnight. The supernatant was discarded, the mixture was washed 5 times with 1 mL of co‐IP wash buffer, the agarose bead‐protein complex was resuspended in 50 µL of 2×SDS loading buffer, and the mixture was heated at 95 °C for 8 min for SDS‒PAGE analysis.

### Immunoblotting

The cells were harvested and lysed in RIPA buffer (50 mM Tris‐HCl, pH 8.0; 150 mm NaCl; 1% NP‐40; 1% Triton X‐100; 0.1% SDS; 1% sodium deoxycholate) supplemented with protease inhibitors, sonicated, and heated at 95 °C for 8 min for SDS‒PAGE analysis.

### Protein Purification

The full‐length sequences of USP8, tagged at the N‐terminus with GST, were inserted into the pET‐N‐GST plasmid. The full‐length sequences of MDA5, which were tagged at the N‐terminus with 6 × His, were inserted into the pET‐28A plasmid. The relevant plasmids were transformed into BL21 (DE3) *E. coli*, and IPTG (final concentration of 0.8 mm) was added to induce protein expression overnight at 16 °C. For his‐MDA5, bacteria were harvested and lysed in lysis buffer (50 mm NaH_2_PO4, 300 mm NaCl, and 10 mm imidazole). After sonication and high‐speed centrifugation, the supernatant containing the target proteins was incubated with Ni‐NTA beads (1018244) overnight at 4 °C. Then, the target proteins were eluted with elution buffer (50 mm NaH_2_PO4, 300 mm NaCl, 250 mm imidazole) prior to washing with wash buffer (50 mm NaH_2_PO4, 300 mm NaCl, 20 mm imidazole). The target proteins were further purified on a 30 kDa ultrafiltration column. GST‐USP8 was purified with a BeyGoldTM GST‐tag Purification Kit (Beyotime) according to the manufacturer's instructions.

### Coomassie Blue staining

After electrophoresis, 10 mL of Coomassie blue fast staining solution (G2059) was added to the SDS‒PAGE gel, which was then incubated at room temperature on a shaker for 30 min. After staining, the staining solution was removed, the gel was washed 3 times (15 min/time) with deionized water at room temperature with gentle shaking on a shaker and then images were taken.

### In Vivo Ubiquitination Assays

The cells were treated with MG132 for 4–6 h before being harvested. One percent SDS (100 µL) was added, and the cell sample was incubated at 95 °C for 10 min. The sample was subsequently diluted ten times with lysis buffer, sonicated, and centrifuged, after which the supernatant was collected. The cell supernatant was incubated with Anti‐Myc or S‐protein agarose beads (2 h at RT or overnight at 4 °C), the supernatant was discarded, the mixture was washed five times with 1 ml of lysis buffer, the agarose bead‐protein complex was resuspended in 50 µL of 2×SDS loading buffer, and the mixture was heated at 95 °C for 8 min for western blotting to detect ubiquitin modification of target proteins. For analysis of ubiquitination by Ni‐IDA, His‐Ub stably transfected HEK293 cells were transfected with the specified vector for 48 h and lysed under denaturing conditions (buffer 1: 8 M Urea; 0.01 M Tris‐HCl, pH 8.0; 0.1 M Na_2_HPO4/NaH_2_PO4; 6 M guanidine‐HCl; 10 mm beta‐mercaptoethanol; 15 mm imidazole) with protease inhibitors. After ultrasonic treatment, the polyubiquitinated proteins were purified by incubating BeaverBeads IDA‐Nickel (Ni‐IDA) at room temperature for 4 h. The pull‐down products were sequentially washed once in buffer 1, once in buffer 2 (buffer 1 without imidazole), once in buffer 3 plus (8 m Urea; 0.01 m Tris‐HCl, pH 6.3; 0.1 m Na_2_HPO4/NaH_2_PO4; 10 mm beta‐mercaptoethanol; 0.2% Triton X‐100), once in buffer 3 (8 m Urea; 0.01 m Tris‐HCl, pH 6.3; 0.1 m Na_2_HPO4/NaH_2_PO4; 10 mm beta‐mercaptoethanol; 20 mm imidazole), and once in buffer 3 plus. Polyubiquitinated proteins were added to 2× sample buffer and separated by SDS‒PAGE for western blot analysis.

### In Vitro Ubiquitination Assays

SFB‐MDA5 and HA‐Ub were co‐transfected into HEK293T cells, or SFB‐MDA5 was transfected into stable His‐Ub‐overexpressing HEK293T cells. The cells were collected and treated according to the aforementioned in vivo ubiquitination assay steps. The S‐protein agarose‐protein complex was eluted with a biotin elution buffer to obtain a solution containing SFB‐MDA5‐Ub. USP8 and USP8 C786A were obtained via in vitro translation kits from TNT Quick Coupled Transcription/Translation Systems (L1170) according to the instructions and verified via western blotting. SFB‐GFP, SFB‐USP8, and USP8 S718A were purified from HEK293T cells. A certain amount of SFB‐MDA5‐Ub solution was incubated with in vitro translated USP8, USP8 C786A, or S718A at 37 °C for 2 h. Then, 5–10 µµm ATP was added, and the mixture was mixed well and incubated overnight at 16 °C. The reaction mixture was used for western blotting to detect ubiquitin modifications of target proteins.

### DUB Assays

For analysis of USP8 activity in the hydrolysis of Di‐Ub chains (AQUApure Di‐Ub Chains (K6‐linked) Protein, UC‐11B‐025, CF R&D Systems), the purchased Di‐Ub chains were prediluted to 0.3 µg µL in 2× DUB buffer (100 mm NaCl; 100 mm Tris‐HCl, pH 7.5; 10 mm DTT) and incubated at room temperature for 15 min. The mixture was evenly mixed with purified SFB‐USP8 from HEK293T cells. The mixture was incubated at 37 °C for 0–3 h or at 16 °C overnight. The reaction mixture was added to 2×Tricine SDS sample buffer and boiled for 5 min for Tris‒Tricine SDS‒PAGE analysis.

### Statistical Analysis

Unless otherwise noted, all experiments were independently repeated at least three times and were analyzed as the mean ± SEM. T‐test, one‐way ANOVA, and two‐way ANOVA were used to evaluate the statistical significance. *p* < 0.05 was considered statistically significant (^*^ *p *< 0.05, ^**^ *p *< 0.01, ^***^ *p *< 0.01). The Kaplan–Meier method was used for mouse survival analysis. Pearson correlation was used with a one‐tailed and 95% confidence interval. GraphPad Prism 9.0 was used for data analysis. Adobe Illustrator CC 2020 was used for image arrangement (Table S7, Supporting Information).

### Ethics

This paper describes studies involving human blood, and all the samples were approved by the Medical Ethics Committee of Tongji Medical College, Huazhong University of Science and Technology (approval number: S170). All animal experiments were approved by the Ethics Committee of Tongji Medical College, Huazhong University of Science and Technology, China (approval number: S2927).

## Conflict of Interest

P.Z., Q.Z., and S.H. are co‐inventors of a patent (ZL202411570947.6) submitted by the Sichuan Academy of Medical Sciences & Sichuan Provincial People's Hospital that covers the functions of USP8 and its inhibitor in autoimmune diseases. All other authors declare that they have no competing interests.

## Author Contributions

Q.Z., S.H., Y.H., and W.W. contributed equally to this work. Q.Z. and P.Z. performed Conceptualization. C.T., M.M., M.Z., L.Y., K.P.K. performed Methodology. Q.Z., S.H., Y.H., W.W. Investigation, Visualization. W.W., K.P.K., P.Z. Resources. Q.Z., P.Z. Funding acquisition, wrote the original draft. QZ, SH Project administration. PZ Supervision, wrote, reviewed, and edited.

## Supporting information



Supporting Information

## Data Availability

The data that support the findings of this study are available from the corresponding author upon reasonable request.
