# Supplementary material for: USP8‐Governed MDA5 Homeostasis Promotes Innate Immunity and Autoimmunity
Source: Adv Sci (Weinh). 2025 Jun 17;12(34):e03865. doi: 10.1002/advs.202503865 (PMC12442633; doi:10.1002/advs.202503865)
Supplement: Supplementary file 1 — Supporting Information [file ADVS-12-e03865-s001.docx]

**Supporting Information**

**USP8-governed MDA5 homeostasis promotes innate immunity and autoimmunity**

Qimin Zhang^1,2†*^, Shan Huang^3†^, Yan He^2†^, Weiwei Wang^4†^, Chao Tong^2^, Mengru Ma^2^, Manyu Zhao^2^, Lian Yi^2^, Klaus-Peter Knobeloch^5^, Peijing Zhang^1,2*^

**Supplementary Information**

**USP8-governed MDA5 homeostasis promotes innate immunity and autoimmunity**

**Figure S1-S8: Pages 3-11**

**Table S1-S7: Pages 12-26**

**Fig. S1-S8**

**Fig. S1**


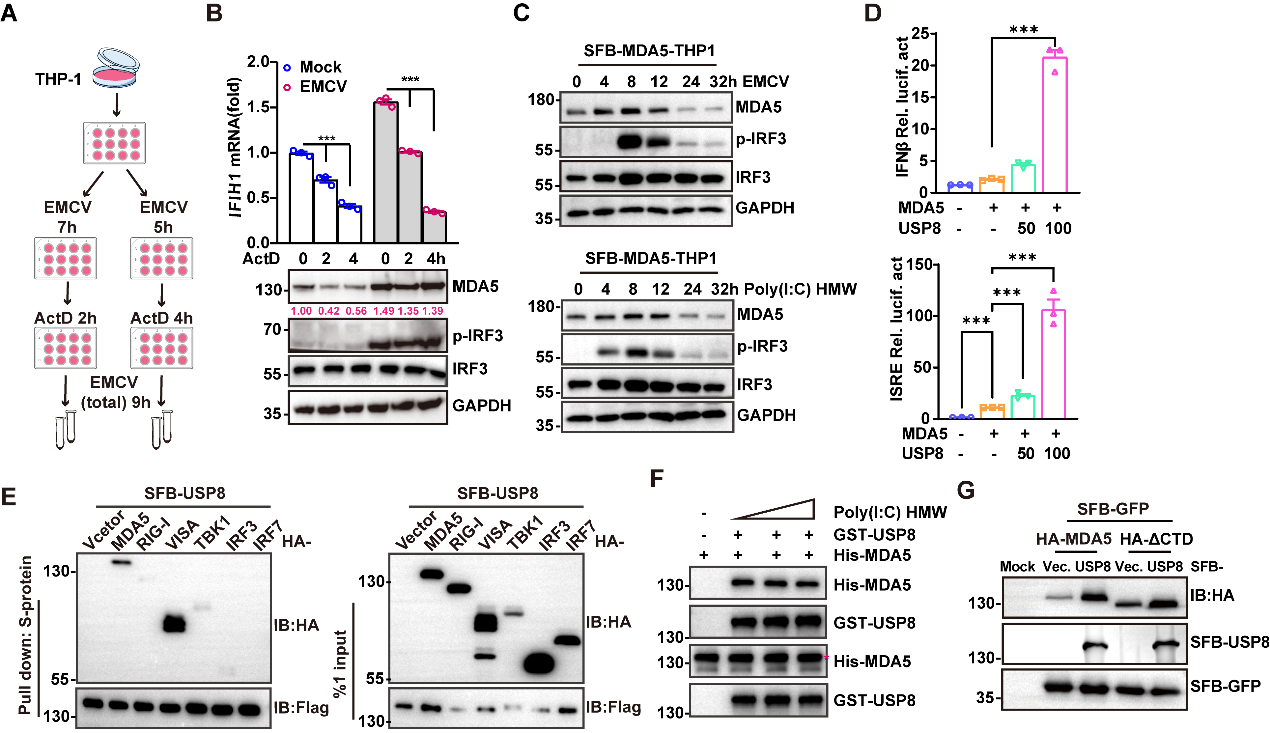


**Fig. S1 Identification of USP8 as an associated protein and regulator of MDA5**

(**A** and **B**) RT‒qPCR and immunoblot analysis of the mRNA and protein levels of MDA5 in THP1 cells infected with or without EMCV after treatment with Actinomycin D (an RNA synthesis inhibitor) at the indicated times, as described in (A). **(C)** Immunoblot analysis of the SFB-MDA5 protein in THP1 cells stably overexpressing SFB-MDA5 infected with EMCV or transfected with Poly(I:C) HMW. **(D)** Luciferase assay in HEK293T cells co-transfected with the IFNβ or ISRE luciferase reporter, pRL-TK, MDA5, or empty vector or DUBs for 24 h. Luciferase activities of IFNβ or ISRE luciferase reporter were normalized to Renilla luciferase. **(E)** Pull-down analysis of the interaction of USP8 with MDA5, RIG-I, VISA, TBK1, IRF3 or IRF7 in HEK293T cells. **(F)** Pull-down analysis of the interaction of USP8 with MDA5 supplementation with 0-1ug/ml Poly(I:C) HMW. **(G)** Immunoblot analysis of wild-type MDA5 or the truncated mutant by overexpression of USP8 in HEK293T cells. The data from the indicated wells per group are presented as the means ± SEMs. Each blot data is representative of three independent experiments. (B and D) *n* = 3 biologically independent experiments. P values were determined via two-way ANOVA (B) and one-way ANOVA (D). ***P<0.001.

**Fig. S2**


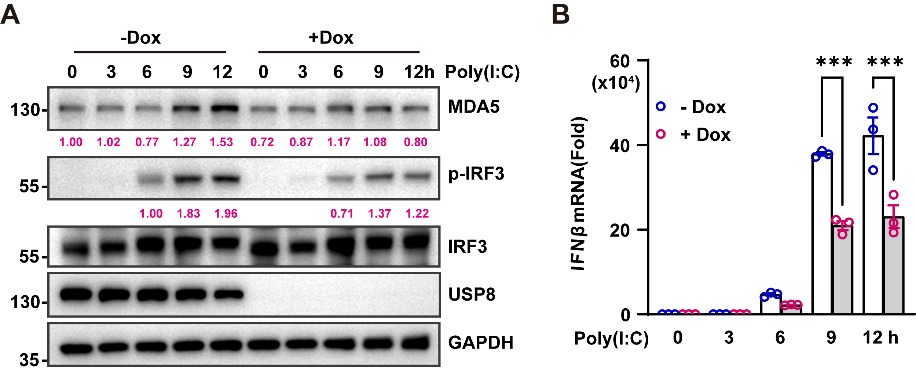


**Fig. S2 USP8 deficiency impairs the poly(I:C)-induced IFN response in vitro**

**(A)** Immunoblot analysis of p-IRF3, p-TBK1, USP8 and GAPDH protein levels in Tet-on pLKO-shUSP8- A549 cells treated -/+ Dox for 72 h followed by transfection with poly(I:C) at the indicated times. **(B)** RT‒qPCR analysis of IFNβ mRNA in Tet-on pLKO-shUSP8- A549 cells treated as in (A). The data from the indicated wells per group are presented as the means ± SEMs. Each blot data is representative of three independent experiments. (B) *n* = 3 biologically independent experiments. P values were determined via two-way ANOVA (B). ***P<0.001.

**Fig. S3**


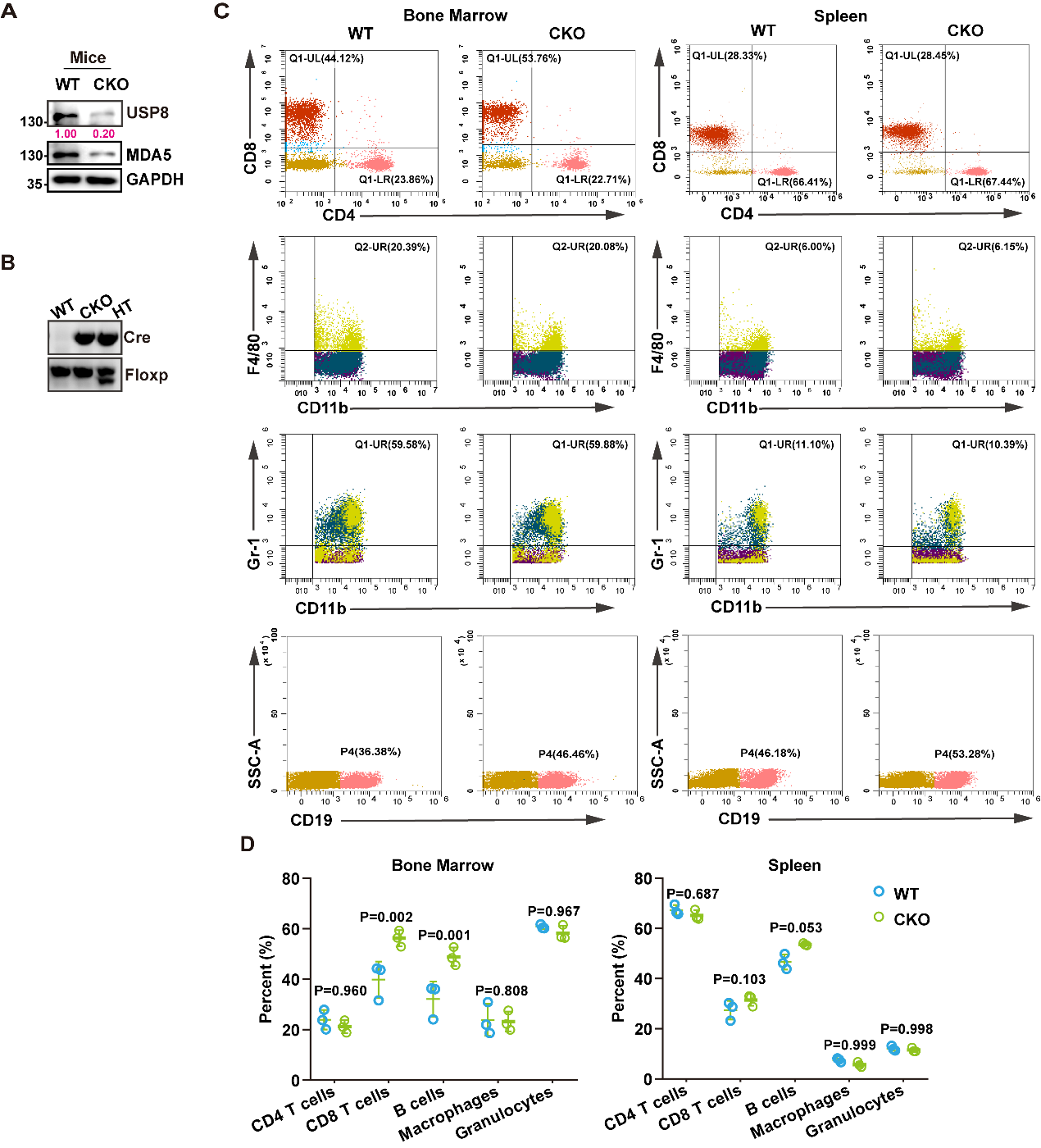


**Fig. S3 Identification of the development of normal immune cells in Usp8 conditional knockout mice**

**(A)** Immunoblot analysis of USP8, MDA5 and GAPDH in the BMDMs of control Usp8^fl/fl^ (WT) and Usp8^fl/fl^ Lyz2-Cre (CKO) mice. **(B)** PCR analysis of Cre and floxp in Usp8^fl/fl^ (WT), Usp8^fl/-^ Lyz2-Cre (HT) and Usp8^fl/fl^ Lyz2-Cre (CKO). **(C-D)** Flow cytometric analysis of the frequency of CD4^+^/CD8^+^ T cells (gated on CD3^+^ cells), macrophages (CD11b^+^ F4/80^+^), granulocytes (CD11b^+^ Gr-1^+^) and B cells (CD19^+^) from the bone marrow and spleen of control Usp8^fl/fl^ (WT) and Usp8^fl/fl^ Lyz2-Cre (CKO) mice. The data from the indicated wells per group are presented as the means ± SEMs. Each blot data is representative of three independent experiments. (D) *n* = 3 per group. P values were determined via two-way ANOVA (D).

**Fig. S4**


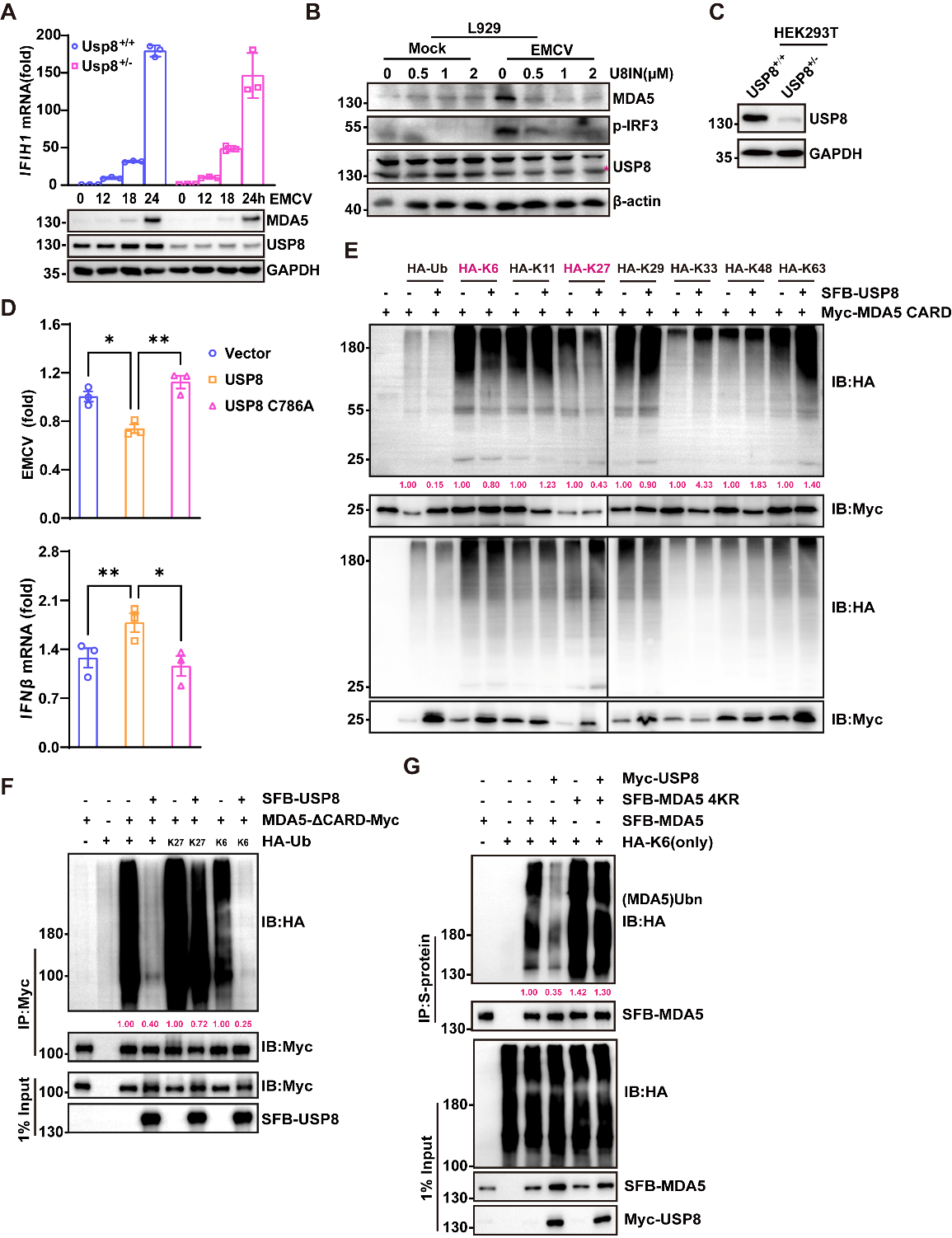


**Fig. S4 The antiviral effect of USP8 depends on its catalytic enzyme activity**

**(A)** Expression of IFIH1 mRNA and immunoblot analysis of MDA5, USP8 and GAPDH in USP8^+/+^ or USP8^+/-^ A549 cells infected with EMCV for 0–24 h. **(B)** Immunoblot analysis of the MDA5, p-IRF3, USP8 and β-actin proteins in L929 cells with or without U8IN (DUBs-IN-2) for 1 h followed by infection with EMCV for 8 h. USP8 was marked with “*”. **(C)** Immunoblot analysis of the USP8 protein in USP8^+/+^ and USP8^+/-^ HEK293T cells. **(D)** RT‒qPCR analysis of EMCV replication and IFNβ mRNA in USP8^+/-^ HEK293T cells transfected with control empty vector, USP8 WT or USP8 C786A for 24 h followed by infection with EMCV for 8 h. **(E)** Denature deubiquitination and immunoblot analysis of HEK293T cells transfected with MDA5-CARD-Myc or HA-Ub (WT, K6 only, K11 only, K27 only, K29 only, K33 only, K48 only or K63 only) with or without SFB-USP8 for 24 h. **(F)** Denaturing deubiquitination and immunoblot analysis of HEK293T cells transfected with MDA5-∆CARD-Myc or HA-Ub (WT, K6 only and K27 only) with or without Myc-USP8 for 24 h. **(G)** Denature immunoprecipitation (with anti-S protein) and immunoblot analysis (with anti-Flag, anti-HA or anti-Myc) of HEK293T cells transfected with SFB-MDA5 4KR (K235R/K498R/K688R/K865R) and empty vector or Myc-USP8, and HA-K6 only for 24h. Data represent analysis of the indicated *n* wells per group, means ± SEMs. Each blot data is representative of three independent experiments. (A, D) *n* = 3 biologically independent experiments. P values were determined via two-way ANOVA (A) and one-way ANOVA (D). *P<0.05, **P<0.01.

**Fig. S5**


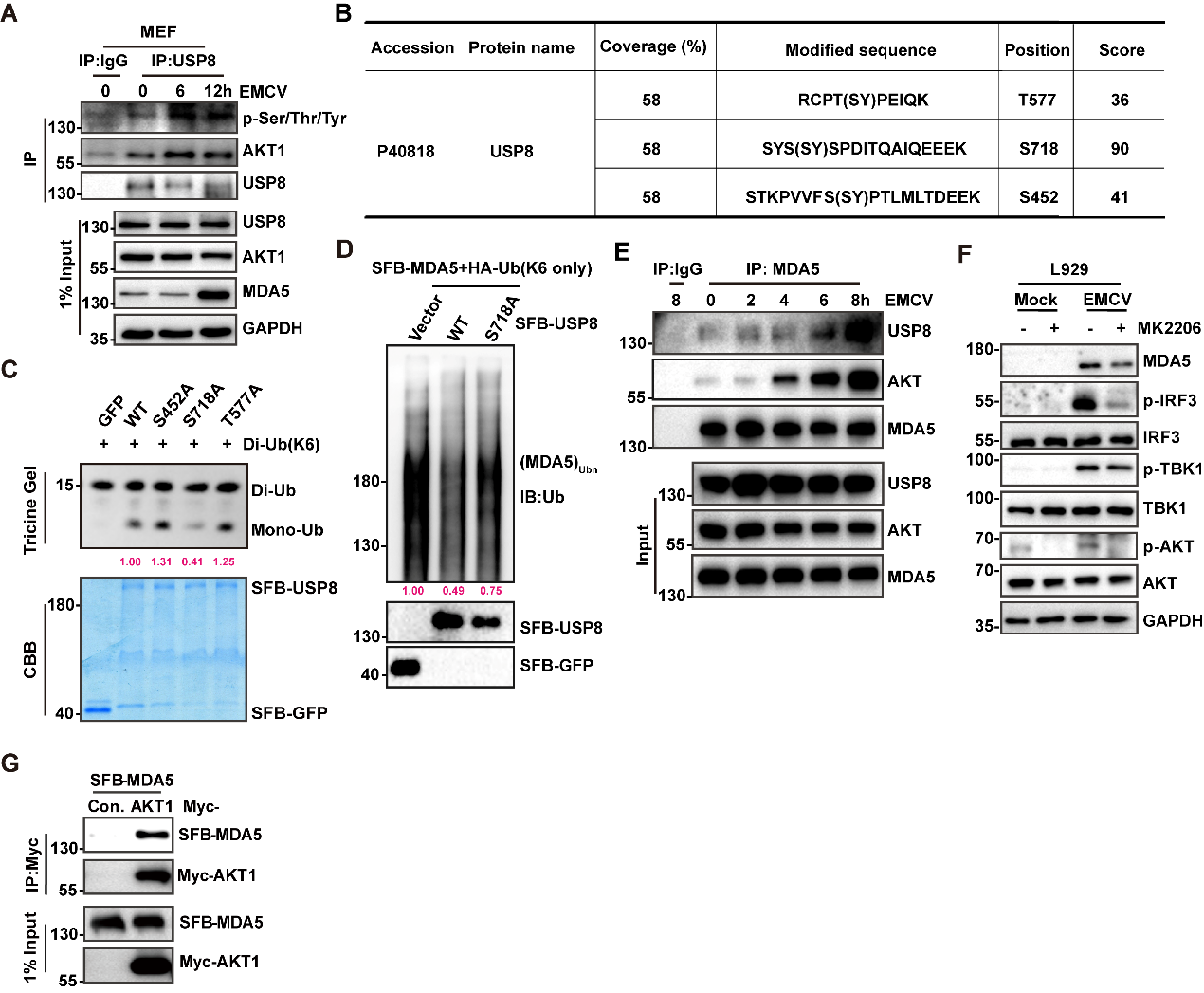


**Fig. S5 AKT1 interacts with and upregulates MDA5 by phosphorylation of USP8**

**(A)** Immunoprecipitation (with anti-USP8) and immunoblot analysis (with anti-p-Ser/Thr/Tyr, anti-AKT1, or anti-USP8) of the phosphorylation levels of endogenous USP8 in MEFs infected with EMCV for 0–12 h. **(B)** Mass spectrometry analysis revealed the potential phosphorylated residues of USP8 in HEK293T cells. **(C)** Purified USP8 and its phosphorylation site mutants were incubated with K6-linked Di-ubiquitin at 37 °C for 2 h. **(D)** In vitro deubiquitination analysis of purified ubiquitin-modified SFB-MDA5 incubated with purified SFB-USP8 and S718A. **(E)** Co-immunoprecipitation analysis of the interaction between MDA5, USP8 and AKT in MDA5 stable expression HEK293T cells infection with EMCV for 0–8 h. **(F)** Immunoblot analysis of the MDA5, p-TBK1, p-IRF3, p-AKT and GAPDH proteins in L929 cells with or without MK2206 treatment for 1 h followed by infection with EMCV for 6 h. **(G)** Co-immunoprecipitation analysis of the interaction between MDA5 and AKT1 in HEK293T cells co-transfected with HA-AKT1 and empty vector or SFB-MDA5. Each blot data is representative of three independent experiments.

**Fig. S6**


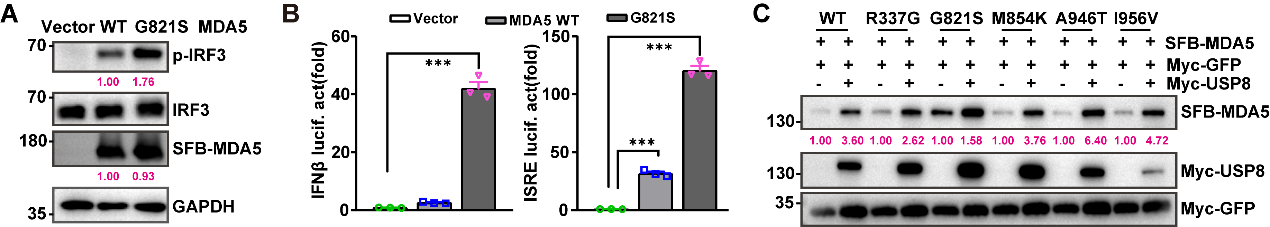


**Fig. S6 USP8 deconjugates K6 ubiquitination of MDA5 at lys235/496/688/865**

**(A)** Immunoblot analysis of MDA5, p-IRF3 and GAPDH. **(B)** Luciferase assay of HEK293T cells co-transfected with IFNβ or ISRE luciferase reporters, pRL-TK, and MDA5 WT or MDA5 G821S for 24 h. Luciferase values were normalized to Renilla luciferase activity. **(C)** Immunoblot analysis of exogenous MDA5 mutants in HEK293T cells transfected with Myc-USP8. Data are representative of two or three independent experiments.The data from the indicated wells per group are presented as the means ± SEMs. Each blot data is representative of three independent experiments. (B) *n* = 3 biologically independent experiments. P values were determined via one-way ANOVA (B). ***P<0.001.

**Fig. S7**


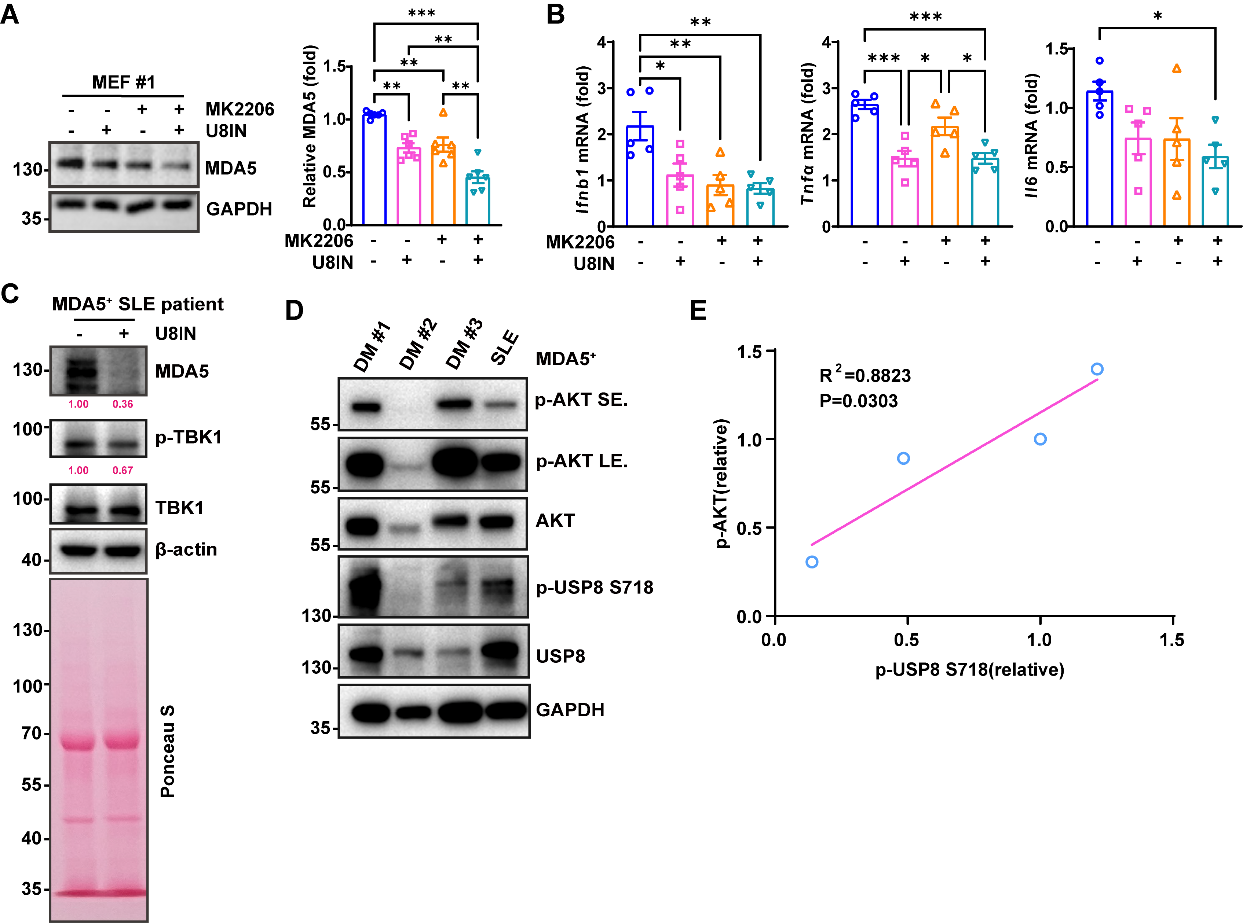


**Fig. S7 Inhibition of USP8 or AKT1 impaired the gain-of-function MDA5 mutant-induced signaling**

**(A)** Immunoblot analysis of MDA5 G821S and GAPDH in gs/+ MEFs treated with U8IN or MK2206 alone or in combination for 9 h. **(B)** RT‒qPCR analysis of Ifnb1, Tnfα and Il6 mRNA in the gs/+ MEFs shown in (A). **(C)** Immunoblot analysis of MDA5 and p-TBK1 in PBMCs from MDA5 SLE patient treated with DMSO or the USP8 inhibitor for 16 h. Ponceau S staining for total protein normalization. **(D)** Immunoblot analysis of p-USP8 and p-AKT in PBMCs from patients. **(E)** Correlation analysis of p-USP8 and p-AKT as shown in (D). The data from the indicated wells per group are presented as the means ± SEMs. Each blot data is representative of six independent experiments. (A) *n* = 6 independent experiments; (B) *n* = 5 per group. P values were determined via one-way ANOVA (A and B) and Pearson correlation with one-tailed and 95% confidence interval (E). *P<0.05, **P<0.01, ***P<0.001.

**Fig. S8**


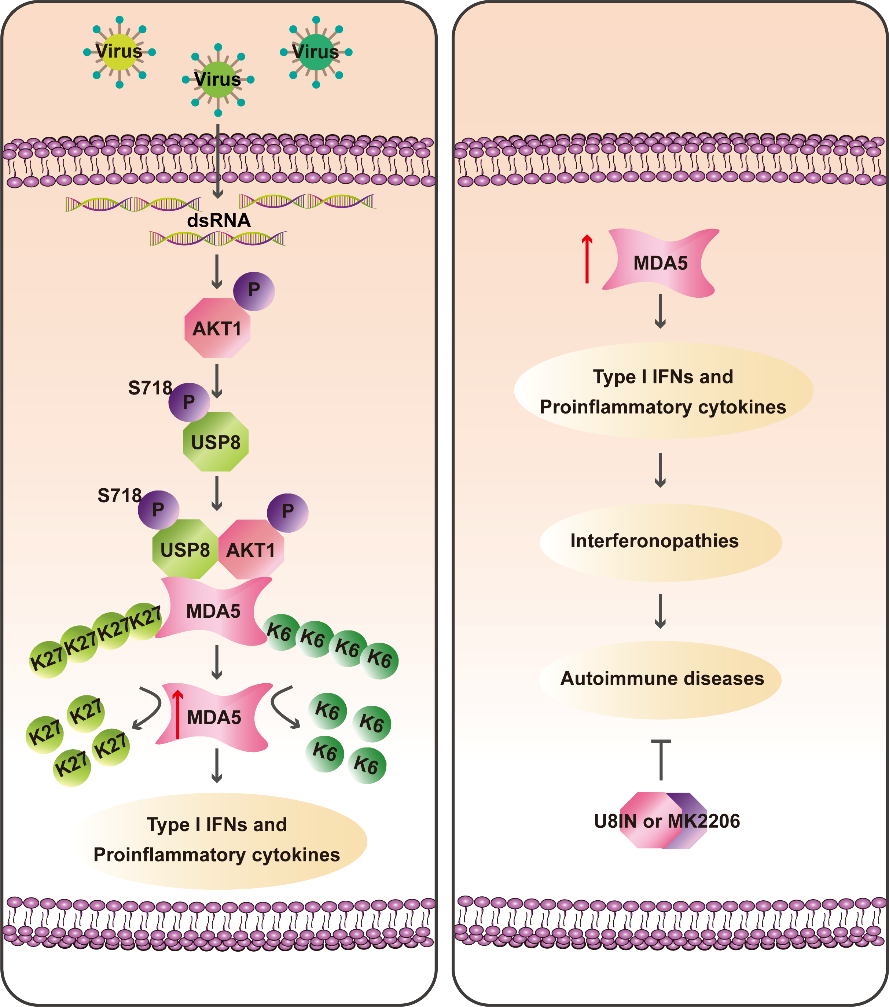


**Fig. S8 Proposed working model of the role of USP8 in the regulation of the antiviral immune response and AGS.**

The homeostasis of the MDA5 protein is controlled by the ubiquitination pathway. Viral infection can activate AKT, resulting in increased enzymatic activity of USP8, which deubiquitinates and stabilizes the MDA5 protein to consequently increase the antiviral immune response. In autoimmune diseases characterized by increased MDA5 activity, USP8 or AKT deficiency suppresses MDA5 mutant-induced interferon and alleviates the symptoms associated with MDA5-related type I interferonopathy.

**Table S1-S7**

**Table S1: Detailed information of Figure 1B**

| **Num.** | **Name** | **Mass(KD)** | **Fold change** | **p value** | **log2(Fc)** | **-log10(p value)** |
| --- | --- | --- | --- | --- | --- | --- |
| 1 | USP1 | 88 | 1.3695625 | 0.0347976 | 0.4537151 | 1.458451 |
| 2 | USP2 | 68 | 1.2697735 | 0.0375325 | 0.3445712 | 1.4255929 |
| 3 | USP3 | 59 | 0.8708552 | 0.1382255 | -0.199495 | 0.8594119 |
| 4 | USP4 | 109 | 0.8314593 | 0.0445718 | -0.266283 | 1.3509394 |
| 5 | USP5 | 96 | 0.3528023 | 9.633E-05 | -1.503068 | 4.0162217 |
| 6 | USP7 | 128 | 0.6771725 | 0.0027607 | -0.562405 | 2.558981 |
| 7 | USP8 | 115 | 4.0718892 | 4.844E-05 | 2.0256983 | 4.3148332 |
| 8 | USP10 | 87 | 1.2128827 | 0.0130287 | 0.2784401 | 1.8850979 |
| 9 | USP11 | 110 | 0.8674961 | 0.0547308 | -0.205071 | 1.261768 |
| 10 | USP12 | 43 | 1.1505418 | 0.168013 | 0.2023134 | 0.774657 |
| 11 | USP13 | 97 | 0.7144413 | 0.003203 | -0.485113 | 2.4944494 |
| 12 | USP14 | 52 | 0.7686468 | 0.000559 | -0.379607 | 3.2525711 |
| 13 | USP15 | 112 | 1.5785496 | 0.0004638 | 0.6585996 | 3.3336282 |
| 14 | USP16 | 94 | 1.7144894 | 0.0061813 | 0.7777789 | 2.2089235 |
| 15 | USP17 | 60 | 1.407393 | 0.3928014 | 0.4930252 | 0.405827 |
| 16 | USP18 | 41 | 0.638006 | 0.0033336 | -0.648358 | 2.4770845 |
| 17 | USP20 | 102 | 0.6977028 | 0.0006862 | -0.519316 | 3.1635335 |
| 18 | USP21 | 63 | 0.8254733 | 0.3194132 | -0.276707 | 0.4956471 |
| 19 | USP22 | 58 | 0.9533372 | 0.5584398 | -0.068942 | 0.2530237 |
| 20 | USP25 | 122 | 0.9815297 | 0.6626348 | -0.026896 | 0.1787258 |
| 21 | USP26 | 104 | 1.2082374 | 0.0031582 | 0.272904 | 2.5005593 |
| 22 | USP28 | 122 | 1.0458091 | 0.2182381 | 0.0646196 | 0.6610695 |
| 23 | USP29 | 104 | 1.2184367 | 0.0043935 | 0.2850313 | 2.3571856 |
| 24 | USP32 | 181 | 1.1586677 | 0.0027144 | 0.2124668 | 2.5663228 |
| 25 | USP33 | 107 | 1.3139771 | 0.103241 | 0.3939402 | 0.9861477 |
| 26 | USP36 | 123 | 1.3172168 | 0.0027706 | 0.3974928 | 2.5574338 |
| 27 | USP37 | 110 | 0.9240628 | 0.1722774 | -0.113937 | 0.7637716 |
| 28 | USP38 | 117 | 0.8023169 | 0.0166158 | -0.317756 | 1.7794785 |
| 29 | USP39 | 65 | 1.5233554 | 0.0007189 | 0.6072526 | 3.1433415 |
| 30 | USP42 | 145 | 1.6478322 | 0.0007411 | 0.7205693 | 3.130126 |
| 31 | USP43 | 123 | 1.0415249 | 0.7016901 | 0.0586974 | 0.1538546 |
| 32 | USP44 | 81 | 1.1498685 | 0.1622747 | 0.2014689 | 0.7897493 |
| 33 | USP45 | 92 | 0.9150285 | 0.4004981 | -0.128111 | 0.3973996 |
| 34 | USP46 | 42 | 1.2291806 | 0.0882291 | 0.2976969 | 1.0543884 |
| 35 | USP47 | 157 | 0.6485759 | 0.0260096 | -0.624653 | 1.5848661 |
| 36 | USP48 | 119 | 0.7445188 | 0.0409921 | -0.42562 | 1.3872993 |
| 37 | USP49 | 79 | 0.9932223 | 0.9357049 | -0.009811 | 0.0288611 |
| 38 | USP50 | 39 | 1.0215006 | 0.8297159 | 0.0306901 | 0.0810706 |
| 39 | USP51 | 80 | 0.8973406 | 0.3077717 | -0.156272 | 0.5117714 |
| 40 | USP52 | 135 | 0.8493437 | 0.2362736 | -0.23558 | 0.6265848 |
| 41 | USP53 | 121 | 0.7004921 | 0.0017529 | -0.513559 | 2.7562484 |
| 42 | USP54 | 187 | 3.5242692 | 0.0041675 | 1.8173241 | 2.3801213 |
| 43 | CYLD | 107 | 0.5142256 | 0.0007197 | -0.959527 | 3.1428528 |
| 44 | UCHL1 | 25 | 0.446443 | 0.0001526 | -1.163452 | 3.8164675 |
| 45 | UCHL3 | 26 | 1.368065 | 0.008963 | 0.4521368 | 2.0475447 |
| 46 | UCHL5 | 38 | 1.1452136 | 0.0359333 | 0.1956167 | 1.4445032 |
| 47 | TNFAIP3 | 90 | 0.6194006 | 0.0002786 | -0.691055 | 3.5549813 |
| 48 | BAP1 | 80 | 1.6519185 | 1.847E-05 | 0.7241425 | 4.7334636 |
| 49 | MYSM1 | 95 | 2.5478748 | 9.935E-05 | 1.3492944 | 4.0028465 |
| 50 | ZRANB1 | 81 | 0.7929814 | 0.006628 | -0.334641 | 2.1786174 |
| 51 | YOD1 | 38 | 0.3384984 | 0.0028653 | -1.562779 | 2.5428366 |
| 52 | PARP11 | 40 | 0.4520377 | 0.0072686 | -1.145485 | 2.1385494 |
| 53 | DUB3 | 60 | 0.6440434 | 0.0260068 | -0.63477 | 1.5849126 |
| 54 | PSMD7 | 37 | 2.0847076 | 0.0004619 | 1.059845 | 3.3354388 |
| 55 | OTUB1 | 31 | 0.366099 | 0.0118018 | -1.449694 | 1.9280527 |
| 56 | OTUB2 | 27 | 0.1104298 | 0.0012603 | -3.178799 | 2.8995207 |
| 57 | OTUD1 | 51 | 0.2699416 | 0.0057043 | -1.889281 | 2.2437986 |
| 58 | OTUD4 | 124 | 1.3021174 | 0.1085358 | 0.3808595 | 0.9644272 |
| 59 | OTUD7B | 93 | 0.823995 | 0.1723112 | -0.279293 | 0.7636865 |
| 60 | JOSD1 | 23 | 1.3729057 | 0.0251151 | 0.4572325 | 1.6000645 |
| 61 | JOSD2 | 21 | 1.1318331 | 0.4018401 | 0.1786612 | 0.3959467 |
| 62 | EIF3S3 | 40 | 1.3729076 | 0.0005306 | 0.4572345 | 3.2752067 |
| 63 | EIF3S5 | 38 | 1.2579324 | 0.0021456 | 0.3310544 | 2.6684548 |
| 64 | BRCC3 | 36 | 1.2102564 | 0.0333635 | 0.2753127 | 1.4767288 |
| 65 | COPS5 | 38 | 0.894823 | 0.381144 | -0.160326 | 0.4189109 |
| 66 | COPS6 | 36 | 1.1209947 | 0.0743913 | 0.1647795 | 1.1284781 |
| 67 | STAMBP1 | 47 | 1.2344677 | 0.033619 | 0.3038891 | 1.4734155 |
| 68 | VCPIP1 | 134 | 1.6393611 | 0.02525 | 0.7131337 | 1.5977387 |

**Table S2: Detailed information of Figure 1C**

| **Num.** | **Name** | **Mass(KD)** | **Fold change** | **p value** | **log2(Fc)** | **-log10(p value)** |
| --- | --- | --- | --- | --- | --- | --- |
| 1 | USP1 | 88 | 0.6146815 | 0.0005096 | -0.702089 | 3.2927421 |
| 2 | USP2 | 68 | 0.9333058 | 0.8409638 | -0.099578 | 0.0752227 |
| 3 | USP3 | 59 | 2.1727763 | 4.449E-05 | 1.1195397 | 4.3517494 |
| 4 | USP4 | 109 | 2.5440849 | 7.7E-05 | 1.3471468 | 4.1134885 |
| 5 | USP5 | 96 | 0.4540674 | 0.0001437 | -1.139022 | 3.8425097 |
| 6 | USP7 | 128 | 0.227776 | 7.331E-06 | -2.134312 | 5.1348535 |
| 7 | USP8 | 115 | 12.485427 | 5.494E-06 | 3.6421733 | 5.260081 |
| 8 | USP10 | 87 | 0.6377851 | 0.0026048 | -0.648858 | 2.5842187 |
| 9 | USP11 | 110 | 3.0444579 | 1.392E-05 | 1.6061854 | 4.8562111 |
| 10 | USP12 | 43 | 0.7846895 | 0.0712112 | -0.349806 | 1.1474516 |
| 11 | USP13 | 97 | 1.152877 | 0.0190293 | 0.2052387 | 1.7205778 |
| 12 | USP14 | 52 | 0.438319 | 7.396E-05 | -1.189947 | 4.1310228 |
| 13 | USP15 | 112 | 0.757455 | 0.0125007 | -0.400768 | 1.9030652 |
| 14 | USP16 | 94 | 0.8997912 | 0.1997391 | -0.152338 | 0.6995368 |
| 15 | USP17 | 60 | 2.2057436 | 9.142E-06 | 1.1412651 | 5.0389355 |
| 16 | USP18 | 41 | 5.0094695 | 0.0001052 | 2.3246578 | 3.9779184 |
| 17 | USP20 | 102 | 3.8339801 | 4.617E-05 | 1.9388428 | 4.3355942 |
| 18 | USP21 | 63 | 0.751776 | 0.0352952 | -0.411625 | 1.4522848 |
| 19 | USP22 | 58 | 1.8856397 | 0.0001645 | 0.9150541 | 3.7838822 |
| 20 | USP25 | 122 | 0.5514389 | 0.0003644 | -0.858727 | 3.4384569 |
| 21 | USP26 | 104 | 1.9294112 | 0.0024729 | 0.9481606 | 2.606799 |
| 22 | USP28 | 122 | 0.6353425 | 0.0003111 | -0.654394 | 3.5071288 |
| 23 | USP29 | 104 | 2.2130439 | 1.052E-06 | 1.146032 | 5.9781198 |
| 24 | USP32 | 181 | 1.5396943 | 5.725E-05 | 0.622644 | 4.2422432 |
| 25 | USP33 | 107 | 2.3079342 | 4.131E-05 | 1.2066021 | 4.3839113 |
| 26 | USP36 | 123 | 1.674464 | 2.103E-05 | 0.7436993 | 4.6772636 |
| 27 | USP37 | 110 | 1.6747146 | 0.0001034 | 0.7439152 | 3.9856629 |
| 28 | USP38 | 117 | 1.0979161 | 0.0145999 | 0.1347678 | 1.8356508 |
| 29 | USP39 | 65 | 0.3749976 | 2.344E-05 | -1.415047 | 4.6299799 |
| 30 | USP42 | 145 | 3.5846692 | 5.348E-06 | 1.84184 | 5.271776 |
| 31 | USP43 | 123 | 3.9055097 | 0.0002186 | 1.9655108 | 3.6603928 |
| 32 | USP44 | 81 | 0.5161308 | 5.775E-05 | -0.954191 | 4.23844 |
| 33 | USP45 | 92 | 0.6878576 | 0.0008668 | -0.539818 | 3.062058 |
| 34 | USP46 | 42 | 0.8134436 | 0.0060586 | -0.297886 | 2.2176265 |
| 35 | USP47 | 157 | 0.4703813 | 0.0001726 | -1.088097 | 3.7629442 |
| 36 | USP48 | 119 | 0.5241713 | 0.0002429 | -0.93189 | 3.6145767 |
| 37 | USP49 | 79 | 0.7607265 | 0.0346091 | -0.39455 | 1.46081 |
| 38 | USP50 | 39 | 1.5378386 | 0.0276795 | 0.6209041 | 1.5578416 |
| 39 | USP51 | 80 | 0.6515245 | 0.0017737 | -0.618109 | 2.7511268 |
| 40 | USP52 | 135 | 1.0140381 | 0.5125113 | 0.0201118 | 0.2902966 |
| 41 | USP53 | 121 | 1.094644 | 0.1885794 | 0.1304618 | 0.7245057 |
| 42 | USP54 | 187 | 10.421473 | 6.159E-05 | 3.3814874 | 4.2104957 |
| 43 | CYLD | 107 | 0.2770225 | 8.306E-06 | -1.851925 | 5.0805984 |
| 44 | UCHL1 | 25 | 0.2796295 | 8.077E-06 | -1.838412 | 5.0927537 |
| 45 | UCHL3 | 26 | 0.5128252 | 4.549E-05 | -0.963461 | 4.342076 |
| 46 | UCHL5 | 38 | 0.7182059 | 0.0038251 | -0.477531 | 2.4173591 |
| 47 | TNFAIP3 | 90 | 1.4250473 | 0.0023739 | 0.5110098 | 2.6245353 |
| 48 | BAP1 | 80 | 2.4590532 | 1.148E-05 | 1.2981029 | 4.9400502 |
| 49 | MYSM1 | 95 | 0.6380261 | 0.0009923 | -0.648313 | 3.0033664 |
| 50 | ZRANB1 | 81 | 0.5628128 | 0.0185382 | -0.829273 | 1.7319331 |
| 51 | YOD1 | 38 | 0.5705552 | 0.0008438 | -0.809562 | 3.0737372 |
| 52 | PARP11 | 40 | 1.4891139 | 0.0006957 | 0.5744541 | 3.1576057 |
| 53 | DUB3 | 60 | 3.8088579 | 8.734E-05 | 1.9293585 | 4.0587628 |
| 54 | PSMD7 | 37 | 2.4253656 | 0.0001329 | 1.2782022 | 3.8764658 |
| 55 | OTUB1 | 31 | 0.9420594 | 0.9022723 | -0.08611 | 0.0446624 |
| 56 | OTUB2 | 27 | 0.9259778 | 0.6700273 | -0.110951 | 0.1739075 |
| 57 | OTUD1 | 51 | 3.1874941 | 3.466E-06 | 1.6724227 | 5.4601508 |
| 58 | OTUD4 | 124 | 7.4948576 | 0.0005722 | 2.9059011 | 3.2424214 |
| 59 | OTUD7B | 93 | 6.6718614 | 1.98E-05 | 2.7380893 | 4.7033695 |
| 60 | JOSD1 | 23 | 1.0100083 | 0.1393905 | 0.0143671 | 0.855767 |
| 61 | JOSD2 | 21 | 3.6394547 | 0.0009111 | 1.8637223 | 3.0404186 |
| 62 | EIF3S3 | 40 | 0.5797008 | 9.129E-05 | -0.78662 | 4.0395885 |
| 63 | EIF3S5 | 38 | 0.6507375 | 0.0077674 | -0.619852 | 2.1097241 |
| 64 | BRCC3 | 36 | 0.8604086 | 0.2785089 | -0.216906 | 0.555161 |
| 65 | COPS5 | 38 | 0.8971183 | 0.4573769 | -0.15663 | 0.3397258 |
| 66 | COPS6 | 36 | 0.6075343 | 0.0040083 | -0.718962 | 2.3970374 |
| 67 | STAMBP1 | 47 | 27.256238 | 2.909E-05 | 4.7685146 | 4.5362079 |
| 68 | VCPIP1 | 134 | 3.1532004 | 0.0006403 | 1.6568169 | 3.1935827 |

**Table S3: Detailed antibodies used in this study**

| Antibodies | Source | Catalog No. |
| --- | --- | --- |
| Anti–USP8 | Proteintech | Cat#27791-1-AP |
| Anti–HA-Tag | Proteintech | Cat#51064-2-AP |
| Anti–Flag-Tag (DKDDDDK) | Proteintech | Cat#20543-1-AP |
| Anti-GAPDH | Proteintech | Cat#60004-1-Ig |
| Anti-Hsp90 | Proteintech | Cat#60318-1-Ig |
| MDA-5 (D74E4) Rabbit mAb | Cell Signaling Technology | Cat#5321 |
| TBK1/NAK (D1B4) Rabbit mAb | Cell Signaling Technology | Cat#3504 |
| Phospho-TBK1/NAK (Ser172)  (D52C2) XP® Rabbit mAb | Cell Signaling Technology | Cat#5483 |
| IRF-3 (D83B9) Rabbit mAb | Cell Signaling Technology | Cat#4302 |
| Phospho-IRF-3 (Ser396)  (4D4G) Rabbit mAb | Cell Signaling Technology | Cat#4947 |
| Phospho-Akt (Ser473) (D9E)  XP® Rabbit mAb | Cell Signaling Technology | Cat#4060 |
| Akt (pan) (C67E7) Rabbit mAb | Cell Signaling Technology | Cat#4691 |
| Phospho-Ser/Thr/Tyr mAb | Enzo Life Sciences | Cat# ADI-905-522-1 |
| Rabbit (DA1E) mAb IgG  XP® Isotype Control | Cell Signaling Technology | Cat#3900 |
| Mouse Anti-rabbit IgG (Conformation  Specific) (L27A9) mAb | Cell Signaling Technology | Cat#3678 |
| Anti-mouse CD16/32(clone 93) | BioLegend | Cat#101320 |
| Anti-CD45-Percp/Cy5.5(clone 30-F11) | BioLegend | Cat#103132 |
| Anti-CD3-PE/Cy7(clone 17A2) | BioLegend | Cat#100218 |
| Anti-CD8α-PE(clone 53-6.7) | BioLegend | Cat#100707 |
| Anti-CD4-FITC(clone RM4-5) | BioLegend | Cat#100510 |
| Anti-Ly6G/Ly6C-PE(clone RB6-8C5) | BioLegend | Cat#108408 |
| Anti-CD11b-APC(clone M1/70) | BioLegend | Cat#101212 |
| Anti-CD19-FITC(clone 6D5) | BioLegend | Cat#115505 |
| Anti–Myc-mAb | GNI | Cat#GNI4110-MC |
| Anti-β-actin | Santa Cruz | Cat#SC-47778 |
| Ubiquitin Antibody (F-11) | Santa Cruz | Cat#SC-278219 |
| Anti-phosphor-Serine718 hUSP8 antibody | This paper | N/A |

**Table S4: Detailed chemicals and recombinant proteins used in this study**

| Chemicals and recombinant proteins | Source | Catalog No. |
| --- | --- | --- |
| HiPure Total RNA Mini Kit | Magen | Cat#R4111-03 |
| cDNA Reverse Transcription Reagent Kit | TakaRa | Cat#RR036A-1 |
| GoScript™ Reverse Transcription System | Promega | Cat#A5001 |
| TnT Quick Coupled Transcription/  Translation Systems | Promega | Cat#L1170 |
| QuantiNova SYBR Green PCR Kit (500) | QIAGEN | Cat#208054 |
| Mouse IFN-β Quantikine ELISA Kit | R&D Systems | Cat# MIFNBO |
| AQUApure Di-Ub Chains  (K6-linked) Protein, CF | R&D Systems | Cat#UC-11B-025 |
| PAGE Gel Fast Preparation Kit (7.5%) | Epizyme | Cat#PG111 |
| Tris-Tricine-SDS‒PAGE Kit | Solarbio | Cat# P1320 |
| Tris-Tricine-SDS‒PAGE  Loading buffer,2×(with DTT) | Solarbio | Cat# P1325 |
| Tris-Tricine-SDS‒PAGE  Electrode buffer,10x (Anode Buffer) | Solarbio | Cat# T1220 |
| Tris-Tricine-SDS‒PAGE  Electrode buffer,10x (Cathode Buffer) | Solarbio | Cat# T1210 |
| Lipofectamine™ 3000 Transfection Reagent | ThermoFisher | Cat#L3000015 |
| Lipo8000^TM^ Transfection Reagent | Beyotime Biotechnology | Cat# C0533 |
| Ponceau S Solution | Servicebio | Cat#G2011 |
| Polyethylenimine, line | Polyscience, Inc | Cat#23966 |
| Recombinant Murine GM-CSF | Peprotech | Catg#315-03 |
| Recombinant Murine IL-4 | Peprotech | Cat#214-14 |
| Tamoxifen | Sigma Aldrich | Cat#T5648 |
| 4-Hydroxytamoxifen | Sigma Aldrich | Cat#H6278 |
| Poly(I:C) HMW | InvivoGen | Cat#tlrl-pic |
| Poly(I:C) | Sigma Aldrich | Cat# 42424-50-0 |
| Chemicals and recombinant proteins | **Source** | Catalog No. |
| MK-2206 2HCl | Selleck | Cat#1032350-13-2 |
| MG132 | Selleck | Cat#S2619 |
| Cycloheximide | Selleck | Cat#S6418 |
| IPTG | Solarbio | Cat#I8070 |
| Doxycycline Hydrochloride | Sigma Aldrich | Cat#D3072 |
| USP8 inhibitor | Meilunebio | Cat# MB7295 |
| Actinomycin D | MCE | Cat#HY-17559 |

**Table S5: Primers used in this study**

| Oligonucleotides | Source | Identifier |
| --- | --- | --- |
| PrimerTime qPCR assay (*GAPDH*):  F 5’- TCCCTGAGCTGAACGGGAAG -3’  R 5’- GGAGGAGTGGGTGTCGCTGT-3’ | This study | N/A |
| PrimerTime qPCR assay (*IFIH1*):  F 5’- GGGGCATGGAGAATAACTCA -3’  R 5’- TGCCCATGTTGCTGTTATGT-3’ | This study | N/A |
| PrimerTime qPCR assay (*CXCL10*): F 5’- GGTGAGAAGAGATGTCTGAATCC -3’  R 5’- GTCCATCCTTGGAAGCACTGCA-3’ | This study | N/A |
| PrimerTime qPCR assay (*ISG56*):  F 5’- TCAGGTCAAGGATAGTCTGGAG -3’  R 5’- AGGTTGTGTATTCCCACACTGTA-3’ | This study | N/A |
| PrimerTime qPCR assay (*IFNβ*): F 5’- CAGTCTGCACCTGAAAAGATATTATG -3’  R 5’-GATTTCCACTCTGACTATGGTCCAGG -3’ | This study | N/A |
| PrimerTime qPCR assay (*USP8*): F 5’- AAGGAGCAATCACAGCAAAGG -3’  R 5’- CTGCATTCTTCGAGCATCCATTA-3’ | This study | N/A |
| PrimerTime qPCR assay (*Gapdh*):  F 5’- TGACCACAGTCCATGCCATCAC -3’  R 5’- GTCAGATCCACGACGGACACAT-3’ | This study | N/A |
| PrimerTime qPCR assay (*Actin*):  F 5’- CCGCGAGCACAGCTTCTTTG -3’  R 5’- GGAGTCCTTCTGACCCATTCC -3’ | This study | N/A |
| PrimerTime qPCR assay (*Usp8*): F 5’- AGACTCTCCGAAAGCCTTAAACT -3’  R 5’- GCCGTTAATCCTTTGGGTTTTGG -3’ | This study | N/A |
| PrimerTime qPCR assay (*Cxcl10*): F 5’- ATCATCCCTGCGAGCCTATCCT -3’  R 5’- GACCTTTTTTGGCTAAACGCTTTC-3’ | This study | N/A |
| PrimerTime qPCR assay (*Ifih1*): F 5’- AGATCAACACCTGTGGTAACACC -3’  R 5’- CTCTAGGGCCTCCACGAACA-3’ | This study | N/A |
| PrimerTime qPCR assay (*Ifnb1*):  F 5’- CAGCTCCAAGAAAGGACGAAC -3’  R 5’- GGCAGTGTAACTCTTCTGCAT-3’ | This study | N/A |
| PrimerTime qPCR assay (EMCV 3D): 5’- CTGCCTTCGGTGTCGC -3’ R 5’-TGGGTCG  AATCAAAGTTGGAG-3’ | This study | N/A |
| Primer for mutation (MDA5 G821S): F 5’- AGG CCCGTAGTCGAGCCAGAGCTGATG-3’ R 5’- CAGCTCTGGCTCGACTACGGGCCTGGA C-3’ | This study | N/A |
| Primer for mutation (MDA5 R337G): F 5’- GAG TGGAAAAACCGGAGTGGCTGTTTACATTG-3’ R 5’- CAGCCACTCCGGTTTTTCCACTCC CTGTAG-3’ | This study | N/A |
| Primer for mutation (MDA5 M854K): F 5’- CGA GAGAAGATGAAGTATAAAGCTATACATTGT G-3’ R 5’- TATAGCTTTATACTTCATCTTCTC TCGGAAATCA-3’ | This study | N/A |
| Primer for mutation (MDA5 A946T): F 5’- AAG AGAAAACAAAACACTGCAAAAGAAGTG TGC-3’ R 5’- TTCTTT TGCAGTGTTTTGTTT TCTCTTACAATGT-3’ | This study | N/A |
| Primer for mutation (MDA5 K235R): F 5’- GTT CAGCCAAATCTGGAGAGGGAGGTCTGGGGC-3’ R 5’-CTCCATGCCCCAGACCTCCCT CTCCAGATTTG-3’ | This study | N/A |
| Primer for mutation (MDA5 K498R): F 5’- CTG GTGTTGGAGGGGCCACGCGACAAGCCA-3’ R 5’- GTTCTTCAGCTTTGGCTTGTCGCGTG GCC CTCCA-3’ | This study | N/A |
| Primer for mutation (MDA5 K688R): F 5’- ACAATAAAATGTTGAGAAGGCTGGCTGAA-3’ R 5’- GTTTTCAGCCAGCCTTCTCAACAT TTTATT-3’ | This study | N/A |
| Primer for mutation (MDA5 K865R): F 5’-GTT CAAAATATGCGACCAGAGGAGTATGCTC  -3’ R 5’- CTCCTCTGGTCGCATATTTTGA  ACACAATGTA-3’ | This study | N/A |
| Primer for mutation (MDA5 I956V): F 5’- TGC CGACTATCAAGTAAATGGTGAAATCATCTG C-3’ R 5’- ATTTCACCATTTACTTGATAGTCG GCACACTTCT-3’ | This study | N/A |
| Primer for mutation (MDA5 CARD): F 5’- TAG GCGGCCGCCGCCACCATGTCGAATGGGTATTCC-3’ R 5’-CGCGGATCCGCAATCAGAGC CTGTTAACTC -3’ | This study | N/A |
| Primer for mutation (MDA5ΔCARD): F 5’- TAGGCGGCCGCCGCCACCATGGGTCCTCA AGTGGAAGAG -3’ R 5’- CGCGGATCCATCC TCATCACTAAATAAACAGC -3’ | This study | N/A |
| Primer for mutation (USP8 Δ1): F 5’- gatttctgaagaagacttgaccggtATGGACAGGCAGGAGGAAGCAC-3’ R 5’- gggccctctagatgcatgctcg agTCATGTGGCTACATCAGTTAC-3’ | This study | N/A |
| Primer for mutation (USP8 Δ2): F 5’- ATGACG GATAAATATACAACAAATGCTAAGG-3’ R 5’- CATTTGTTGTATATTTATCCGTCATCATT GTG-3’ | This study | N/A |
| Primer for mutation (USP8 Δ3): F 5’- GAG AATGGGACCAATAACAGGAGTAAAAAGAC-3’ R 5’- TACTCCTGTTATTGGTCCCATTCT  CTCATTTTG-3’ | This study | N/A |
| Primer for mutation (USP8 Δ4): F 5’- gatttctgaagaagacttgaccggtATGCCTGCTGTGGCTTCAGTTC-3’ R 5’- gggccctctagatgcatgctcg agTCAAGCTGGTCC AGAACCTCC-3’ | This study | N/A |
| Primer for mutation (USP8 C786A): F 5’- CTT CGTAACTTAGGAAATACTGCATATATGAAC TC-3’ R 5’- TGCAATATTGAGTTCATATATGC AGTATTTCCTAAG-3’ | This study | N/A |
| Primer for mutation (USP8 S452A): F 5’- CAG TAG TTT TTG CTC CAA CTC TCA TGT TAA C-3’ R 5’- TGA GAG TTG GAG CAA AAA CTA CTG GCT TG-3’ | This study | N/A |
| Primer for mutation (USP8 T577A): F 5’- GGA AAA GGT GTC CAG CCC CAG AAA TAC AG-3’ R 5’- TTT CTG TAT TTC TGG GGC TGG ACA CCT TTT C-3’ | This study | N/A |
| Primer for mutation (USP8 S718A): F 5’- AGC GCT CCT ACG CAT CCC CAG ATA TAA CCC AG-3’ R 5’- ATC TGG GGA TGC GTA GGA GCG CTT CAG TTT GG-3’ | This study | N/A |
| Primer for genotyping (Lyz2-Cre mice):  F 5’- CCCAGAAAT GCCAGATTACG -3’  R 5’- CTTGGGCTGCCAGAATTTCTC -3’ | This study | N/A |
| Primer for genotyping (Cre-ER mice):  F 5’- AAAGTCGCTCTGAGTTGTTAT -3’  R 5’- CCTGATCCTGGCAATTTCG -3’ | This study | N/A |
| Primer for genotyping (*Usp8*^fl/fl^ mice):  F 5′-CCATGACTGCCTTTCCAGTT -3’  R 5’- GCGATGATGAAATTGAAATAGAT -3’ | This study | N/A |
| Primer for genotyping (*Ifih1*^gs/+^ mice):  F1 5′- ATAGATGGCTTTACTGTCCAAG -3’  R1 5’- ACAGTCATCTGTGGCAATCAA -3’ | This study | N/A |
| Primer for genotyping (*Ifih1*^gs/+^ mice):  F2 5′- ATGGGGCTAAATGGGTGGTTGTG -3’  R2 5’- CCTGAAGAAGTAGCCACACAGTC -3’ | This study | N/A |
| Primer for mutagenesis (*Ifih1*^gs/+^ mice):  gRNA-1 5’-ATCAGCTCTGGCTCGACCCCGGG-3’  gRNA-2  5’- CATCAGCTCTGGCTCGACCCCGG-3’ | This study | N/A |
| Single-sranded donor:  5’-GAGCTCATAACAAGACACAACACTTCCTTTTTTTGTTGTTGTTCACATTCTCAGGCCCGGTCTCGAGCCAGAGCTGATGAAAGCACGTATGTCCTGGTCACCAGCAGTGGCTCAGGAGTTAC-3’ | This study | N/A |
| Primer for Human USP8-targeted sgRNA:  F 5′- caccgATGCAGATTAGATCGTGATG -3’  R 5’- aaacCATCACGATCTAATCTGCATc -3’ | This study | N/A |

**Table S6: Recombinant DNA used in this study**

| Recombinant DNA | Source | Identifier |
| --- | --- | --- |
| Plasmid: pLKO-shUSP8, guide sequence: 5’- CCGGAATCTTCAGCAGCTTATATCCCTCG AGGGATATAAGCTGCTGAAGATTTTTTTG-3’ | This study | N/A |
| Plasmid: EZ-Tet-pLKO-shUSP8, guide sequence: 5’- CTAGAATCTTCAGCAGCTTATATCCCTC GAGGGATATAAGCTGCTGAAGATTTTTTT G-3’ | This study | N/A |
| Plasmid: LentiV2-Cas9-sgUSP8, guide sequence: 5’- ATGCAGATTAGATCGTGATG-3’ | This study | N/A |
| Plasmid: LentiV2-Cas9-sg*IFIH1*, guide sequence: 5’- TAGCGGAAATTCTCGTCTG-3’ and 5’- CGTCATTGTCAGGCACAGAG-3’ | This study | N/A |
| Plasmid: pSpCas9 BB-2A-sgUSP8 (PX458), guide sequence: 5′‐ATGCAGATTAGATCG TGATG‐3′ | This study | N/A |

**Table S7: Software used in this study**

| Software | Source | URLs |
| --- | --- | --- |
| ImageJ | National Institutes of Health | http://www.imagej.nih.gov/ij/ |
| GraphPad Prism 9.0 | GraphPad Software | http://www.graphpad.com |
| Adobe Illustrator 2020 | Adobe | https://www.adobe.com/products/illustrator.html |
| CytExpert software 2.3 | CytExpert | https://cytexpert.software.informer.com/2.3/ |
